# Supplementary material for: Ortho-Phosphinoarenesulfonamide-Mediated Staudinger Reduction of Aryl and Alkyl Azides
Source: Molecules. 2022 Sep 5;27(17):5707. doi: 10.3390/molecules27175707 (PMC9458194; doi:10.3390/molecules27175707)
Supplement: Supplementary file 1 [file molecules-27-05707-s001.zip › molecules-1865967-supplementary.pdf]

SUPPORTING INFORMATION

ortho-Phosphinoarenesulfonamide-mediated Staudinger reduction of aryl and alkyl azides

Xingzhuo Li [1], Zhenguo Wang [1], Wenjun Luo [1], Zixu Wang [1], Keshu Yin [1], and Le Li [1,2*]

[1] PCFM Lab and GDHPRC Lab, School of Chemistry, Sun Yat-sen University, Guangzhou 510275, China
[2] Center for Nephrology and Clinical Metabolomics and Division of Nephrology and Rheumatology, Shanghai Tenth People's Hospital, School of Medicine, Tongji University, Shanghai 200072, China
* Correspondence: lile26@mail.sysu.edu.cn

Table of Contents

1 DFT Studies and Corresponding Cartesian Coordinates S2

2 NMR Spectra of New Compounds S34

3 Reference List for the Characterization Data of the Azides **1** and the Products **3** S43

1 DFT Studies and Corresponding Cartesian Coordinates

Computational details: The calculations were carried out with the Gaussian 09 software package[34]. The structures were optimized by the density functional theory (DFT)[35] with the B3LYP functional[36–37] with basis set 6-31G(d)[38–39] in the gas phase. Frequency analysis was conducted at the same level of theory to verify the stationary points to be real minima or saddle points and to obtain the thermodynamic energy corrections at 298.15 K. Intrinsic reaction coordinate (IRC)[40–42] calculations were performed to confirm the connection between two correct minima for a transition state. More accurate electronic energy results were refined by calculating the single-point energy at the B3LYP-D3(BJ)[10]/6-311++G(2df, 2p)[38–39] level of theory with the SMD model[44] (solvent = THF).

Table S1. Calculated energy data and imaginary frequencies for all structures.

Structure	Energy (au)	Thermal correction to Enthalpy（au）	Thermal correction to Gibbs Free Energy (au)	Imaginary frequency (cm^{-1})
	RB3LYP-D3(bj) 6-311++G(2df,2p) SMD (THF)	B3LYP 6-31G(d) GAS	B3LYP 6-31G(d) GAS	B3LYP 6-31G(d) GAS
**SM**	-1909.23186094	0.422983	0.333759	None
**PPh_3**	-1036.65233648	0.291106	0.227373	None
**H_2O**	-76.46961273	0.024939	0.002838	None
**4-CO_2Me-PhN_3**	-623.97572241	0.159414	0.108260	None
**TS-1**	-2533.20730189	0.582809	0.463490	None
**TS-1′**	-1660.62244703	0.451061	0.355397	1
**Int-1**	-2533.23343372	0.584702	0.465963	None
**Int-1′**	-1660.65160277	0.452861	0.360644	None
**TS-2**	-2533.19295898	0.582229	0.464798	None
**TS-2′**	-1660.61786043	0.450685	0.359975	1
**Int-2**	-2423.75255325	0.573544	0.460855	None
**Int-2′**	-1551.16866891	0.441744	0.353330	0
**TS-3**	-2423.74126002	0.568899	0.457782	1
**TS-3′**	-1627.60300964	0.465044	0.374102	None
**Int-3**	-2423.74352190	0.572330	0.460629	None
**Int-3′**	-1627.62266367	0.470228	0.380589	None
**Int-4**	-2423.73620712	0.573688	0.461709	None
**Int-4′**	-1627.62640480	0.470250	0.379852	0
**TS-4**	-2423.71898879	0.569503	0.457229	None
**TS-4′**	-1627.62190583	0.468755	0.379071	None
**Product**	-1908.03804540	0.399955	0.313968	0
**Ph_3PO**	-1111.95367790	0.296993	0.232914	None
**N_2**	-109.56124218	0.008904	-0.012851	None
**4-CO_2Me-$PhNH_2$**	-515.72017775	0.171881	0.124814	None

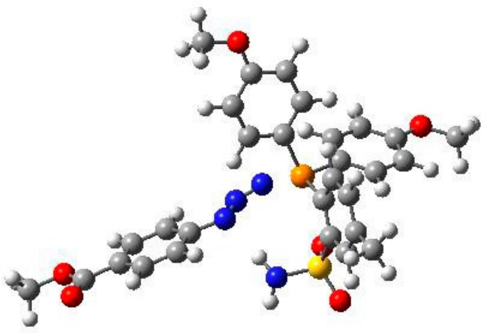

TS-1

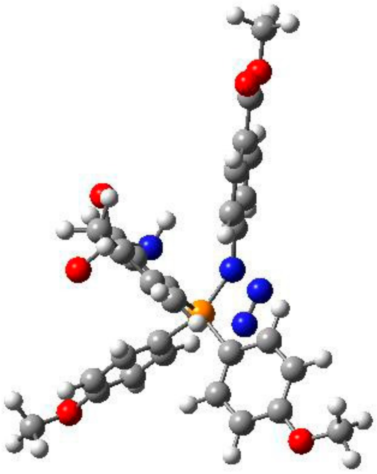

TS-2

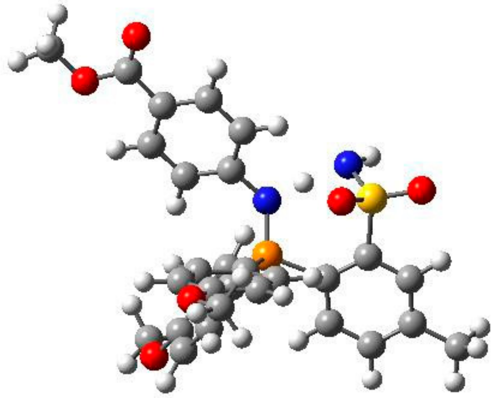

TS-3

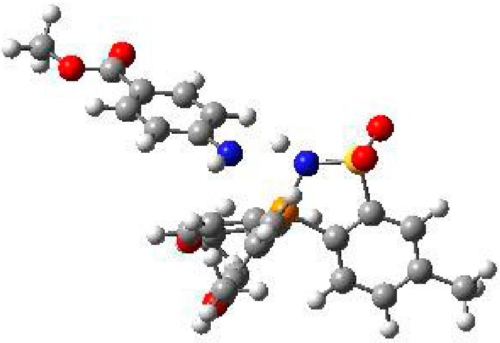

TS-4

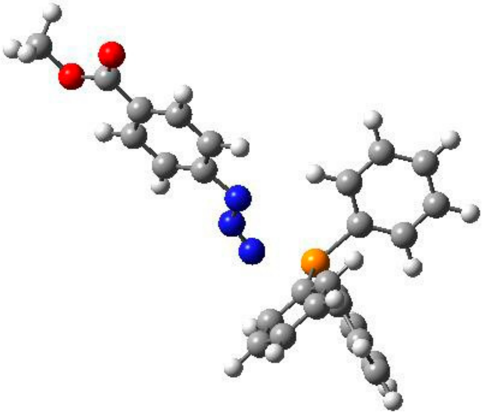

TS-1′

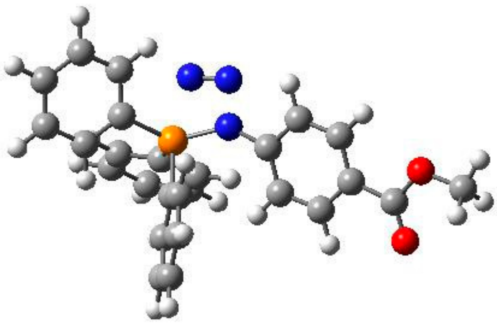

TS-2′

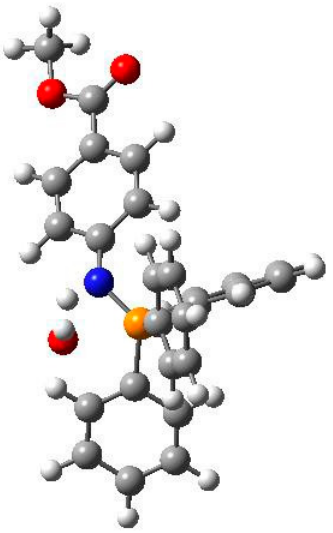

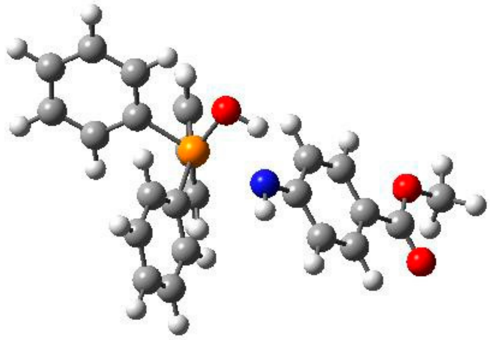

TS-3′ **TS-4′**

Cartesian coordinates for all calculated structure

SM

C	-0.65923	-2.31311	2.36497
C	0.09787	-1.55543	1.46874
C	-0.49816	-0.79324	0.45504
C	-1.90512	-0.85975	0.37497
C	-2.66627	-1.61293	1.26406
C	-2.05237	-2.35544	2.28176
H	-0.15084	-2.88086	3.14101
H	1.17953	-1.5609	1.55836
H	-3.74576	-1.6177	1.15138
S	-2.84763	0.07124	-0.85375
O	-4.27153	-0.23899	-0.6852
O	-2.38052	1.46281	-0.81876
P	0.51625	0.17399	-0.78298
C	0.82792	1.7687	0.08524
C	1.11278	2.88321	-0.71306
C	0.79604	1.94972	1.47947
C	1.38014	4.13593	-0.15786
H	1.11966	2.78033	-1.79538
C	1.05122	3.19073	2.04759
H	0.55841	1.11366	2.13028
C	1.34804	4.29361	1.2325
H	1.59552	4.97303	-0.81206
H	1.02061	3.33501	3.12327

C	2.14622	-0.69417	-0.68453
C	2.32841	-1.8144	-1.50764
C	3.23207	-0.28256	0.1096
C	3.5294	-2.52527	-1.52941
H	1.51604	-2.14554	-2.15101
C	4.43846	-0.97198	0.09065
H	3.13482	0.59104	0.74704
C	4.59514	-2.10206	-0.7252
H	3.62538	-3.38946	-2.1769
H	5.27809	-0.65344	0.70106
C	-2.88216	-3.1776	3.23834
H	-3.65834	-2.56858	3.71635
H	-3.39262	-3.9974	2.71762
H	-2.26383	-3.61688	4.02717
N	-2.40285	-0.67917	-2.30816
H	-3.20172	-0.63287	-2.93954
H	-1.58168	-0.21679	-2.69912
O	1.58305	5.46616	1.88682
O	5.81471	-2.70911	-0.66925
C	6.0328	-3.85852	-1.47281
H	7.05668	-4.1763	-1.26805
H	5.34178	-4.67072	-1.21128
H	5.93146	-3.62915	-2.54172
C	1.86078	6.62394	1.11424
H	1.02511	6.87357	0.44756
H	2.00482	7.43412	1.8313
H	2.77437	6.50169	0.51744

PPh_3

P	-0.00149	0.00827	0.00568
C	-0.00236	0.00059	1.85991
C	1.25125	-0.02909	2.49455
C	-1.15523	0.059	2.65774
C	1.35015	-0.02339	3.88554
H	2.15649	-0.05228	1.8919
C	-1.056	0.07664	4.05085
H	-2.13498	0.0955	2.19094
C	0.19479	0.03166	4.66825
H	2.32875	-0.05104	4.3579
H	-1.95946	0.12469	4.6537
H	0.26977	0.04628	5.75243
C	-1.74003	0.50905	-0.39992
C	-2.80714	-0.38542	-0.57443

C	-1.98116	1.88268	-0.57104
C	-4.08138	0.08303	-0.9018
H	-2.64116	-1.45226	-0.45832
C	-3.25612	2.35188	-0.88702
H	-1.16082	2.58828	-0.46036
C	-4.31001	1.45145	-1.05554
H	-4.89648	-0.62371	-1.03628
H	-3.42408	3.41851	-1.01206
H	-5.30216	1.81387	-1.31166
C	0.00312	-1.79997	-0.40411
C	0.37297	-2.15314	-1.71286
C	-0.3053	-2.82438	0.50411
C	0.41349	-3.48959	-2.10979
H	0.63586	-1.37341	-2.4242
C	-0.25437	-4.16332	0.11009
H	-0.58234	-2.57616	1.52433
C	0.10134	-4.49935	-1.19709
H	0.69878	-3.74271	-3.12766
H	-0.49408	-4.94461	0.82709
H	0.14154	-5.54204	-1.50122

H_2O

H	-0.40071	-2.67002	5.49892
H	0.41377	-1.5515	6.13473
O	0.41958	-2.16684	5.38634

4-CO_2Me-PhN_3

C	0.25491	0.02685	1.74629
C	1.17692	0.34994	2.75194
C	-0.5973	-1.073	1.92356
C	1.24856	-0.40965	3.91404
H	1.8376	1.19926	2.62011
C	-0.53298	-1.83726	3.07992
H	-1.30639	-1.31295	1.1384
C	0.39153	-1.508	4.08116
H	1.96595	-0.15131	4.68798
H	-1.18653	-2.69051	3.22837
C	0.13709	0.80672	0.48535
O	-0.65276	0.5601	-0.40554
O	1.01449	1.83653	0.43064
C	0.95063	2.63115	-0.76341

H	-0.03871	3.08377	-0.87121
H	1.7139	3.4006	-0.64322
H	1.15724	2.01784	-1.6445
N	0.3875	-2.34153	5.22592
N	1.8631	-1.98532	7.05119
N	1.18707	-2.09365	6.13923

TS-1

C	3.74703	0.89881	-2.5671
C	4.76462	0.07484	-2.06065
C	2.52241	0.97396	-1.9218
C	4.53577	-0.66723	-0.89635
C	3.30114	-0.57916	-0.25093
C	2.27981	0.24018	-0.74259
H	5.30066	-1.31659	-0.48654
H	3.14328	-1.16979	0.64494
C	0.45109	2.09466	0.5588
C	1.53003	2.99039	0.68208
C	-0.83978	2.5684	0.83443
C	1.32306	4.30535	1.07581
H	2.5391	2.65654	0.46075
C	-1.05953	3.88639	1.23564
H	-1.6915	1.90107	0.72714
C	0.02622	4.76344	1.35795
H	-2.0717	4.21688	1.43814
P	0.63155	0.34628	0.03221
N	-0.57357	0.42203	-1.87571
N	-1.73423	0.2309	-1.75367
N	-2.53089	-0.11961	-0.7969
H	2.15053	5.00174	1.17003
H	3.93885	1.46315	-3.4742
C	0.78859	-0.57652	1.65033
C	0.49368	-1.94273	1.82186
C	1.20103	0.12154	2.79805
C	0.62995	-2.57796	3.05547
C	1.33247	-0.51083	4.03272
H	1.43178	1.17844	2.72576
C	1.05009	-1.87211	4.18702
H	0.40612	-3.63817	3.11985
H	1.66678	0.06914	4.88991
S	-0.08064	-2.98895	0.46902
O	0.51672	-2.44985	-0.76268
O	0.12452	-4.39553	0.82828

H	1.74482	1.60504	-2.33884
C	-3.9001	-0.14045	-1.11295
C	-4.44321	0.17912	-2.37257
C	-4.76964	-0.51255	-0.07035
C	-5.81521	0.12581	-2.57667
H	-3.7785	0.46341	-3.18222
C	-6.13944	-0.5622	-0.28181
H	-4.34897	-0.75487	0.90143
C	-6.68226	-0.24435	-1.53606
H	-6.22503	0.37101	-3.55038
H	-6.81441	-0.84746	0.51857
C	-8.15339	-0.31715	-1.70203
O	-8.93772	-0.6304	-0.82524
O	-8.55208	0.00744	-2.95867
C	-9.9669	-0.04884	-3.18213
H	-10.11032	0.23701	-4.225
H	-10.34619	-1.05923	-3.00565
H	-10.49118	0.64441	-2.51847
N	-1.73562	-2.72344	0.54596
H	-1.99264	-1.83667	0.08548
H	-2.21784	-3.52082	0.13263
C	1.16693	-2.55439	5.52809
H	1.92665	-2.07666	6.15519
H	0.21575	-2.50913	6.07455
H	1.42832	-3.61154	5.41761
O	5.92655	0.06908	-2.76926
O	-0.06928	6.06677	1.73469
C	-1.35629	6.59661	2.0229
H	-1.82877	6.07116	2.86272
H	-1.19362	7.64059	2.29548
H	-2.01635	6.54805	1.1475
C	6.98572	-0.76569	-2.32353
H	6.68939	-1.82246	-2.31666
H	7.79846	-0.62261	-3.03762
H	7.32939	-0.47964	-1.3208

TS-1′

C	3.83731	0.32825	-2.65321
C	4.8944	-0.07985	-1.83754
C	2.56344	0.50362	-2.11277
C	4.6744	-0.31352	-0.4787
C	3.40223	-0.13506	0.06659
C	2.33515	0.28134	-0.74499

H	5.49303	-0.63695	0.15877
H	3.2374	-0.32636	1.12277
C	0.59988	2.31463	0.43412
C	1.76617	3.05219	0.69672
C	-0.65156	2.93946	0.56192
C	1.67729	4.38609	1.0958
H	2.74112	2.5872	0.58929
C	-0.73364	4.27111	0.96955
H	-1.55025	2.37573	0.32686
C	0.42912	4.99652	1.23573
H	-1.70682	4.7448	1.0662
P	0.63858	0.55485	-0.10412
N	-0.50434	-0.45576	-1.83479
N	-1.66993	-0.34194	-1.63525
N	-2.40129	0.23385	-0.75291
H	2.58527	4.94846	1.29713
H	0.36416	6.03684	1.54327
H	4.00127	0.50118	-3.71333
H	5.88526	-0.22208	-2.26057
C	0.53744	-0.46122	1.42019
C	0.49305	-1.85884	1.27408
C	0.44993	0.09467	2.70465
C	0.38092	-2.68084	2.39347
C	0.33104	-0.7337	3.8223
H	0.47383	1.17202	2.8336
C	0.29855	-2.12015	3.67063
H	0.34924	-3.7597	2.26784
H	0.26482	-0.29155	4.81286
H	1.73871	0.79711	-2.75542
C	-3.78786	0.0881	-0.89477
C	-4.42567	-0.5819	-1.95795
C	-4.57974	0.66967	0.11305
C	-5.81012	-0.66248	-2.0062
H	-3.82226	-1.03279	-2.73961
C	-5.96258	0.58567	0.05885
H	-4.08551	1.17624	0.93665
C	-6.59854	-0.08004	-1.00035
H	-6.29203	-1.17887	-2.82923
H	-6.57675	1.03147	0.83454
C	-8.07878	-0.13599	-1.00501
O	-8.79766	0.35612	-0.15428
O	-8.57202	-0.80686	-2.07866
C	-10.00108	-0.89304	-2.13727
H	-10.22393	-1.45513	-3.04527

H	-10.39401	-1.41228	-1.25861
H	-10.44748	0.10427	-2.18444
H	0.20461	-2.76207	4.54231
H	0.54128	-2.30166	0.28274

Int-1

C	4.09286	0.82398	-1.96828
C	2.87471	1.04975	-1.3264
C	2.09653	-0.00266	-0.82322
C	2.60029	-1.31405	-0.99795
C	3.81397	-1.53069	-1.64231
C	4.58178	-0.46988	-2.14403
H	4.66186	1.67273	-2.33902
H	2.52256	2.07009	-1.22399
H	4.16318	-2.55361	-1.74085
S	1.8243	-2.82448	-0.34337
O	2.78048	-3.928	-0.49606
O	1.33368	-2.48796	1.0003
P	0.50139	0.4463	-0.03154
C	0.70172	0.4715	1.77274
C	-0.44294	0.64083	2.57683
C	1.94837	0.33068	2.3897
C	-0.33149	0.67434	3.95722
H	-1.42194	0.7265	2.11503
C	2.0701	0.36953	3.77869
H	2.84004	0.17412	1.79154
C	0.92638	0.54021	4.56913
H	-1.20569	0.79457	4.5889
H	3.04888	0.25032	4.22807
C	0.11151	2.16247	-0.50405
C	-0.59032	2.38956	-1.69607
C	0.47956	3.26864	0.28459
C	-0.91743	3.68161	-2.10369
H	-0.89835	1.54582	-2.30553
C	0.164	4.55876	-0.11468
H	1.00662	3.11704	1.22162
C	-0.53901	4.77539	-1.31181
H	-1.46906	3.82306	-3.02575
H	0.43803	5.41863	0.48819
N	-0.58628	-0.60885	-0.67939
N	-1.85234	-0.37295	-0.27098
N	-2.6583	-1.25036	-0.71419
C	-4.0043	-1.00576	-0.36628

C	-4.88869	-2.08243	-0.54711
C	-4.51415	0.22393	0.09719
C	-6.24014	-1.95179	-0.25267
H	-4.48398	-3.0193	-0.91737
C	-5.86382	0.35312	0.38765
H	-3.83928	1.06446	0.2126
C	-6.74287	-0.72997	0.21986
H	-6.9127	-2.79129	-0.38911
H	-6.26855	1.29563	0.74287
C	-8.17558	-0.52218	0.54404
O	-8.65409	0.52289	0.94557
O	-8.92234	-1.63863	0.34601
C	-10.31669	-1.49905	0.64561
H	-10.77112	-0.72474	0.02112
H	-10.76108	-2.47224	0.43258
H	-10.46176	-1.2329	1.69641
C	5.88628	-0.73895	-2.85242
H	6.53182	-1.39585	-2.25852
H	5.71414	-1.24062	-3.81278
H	6.43296	0.18755	-3.05192
N	0.61614	-3.09044	-1.46665
H	-0.1372	-2.39611	-1.3192
H	0.30013	-4.05668	-1.3902
O	-0.80557	6.07208	-1.60915
O	0.92647	0.58846	5.92666
C	-1.53921	6.36318	-2.79315
H	-1.64075	7.44906	-2.81892
H	-1.00338	6.02608	-3.68936
H	-2.53528	5.90421	-2.76938
C	2.15962	0.4183	6.61279
H	1.9189	0.47902	7.67528
H	2.60718	-0.5597	6.39583
H	2.87471	1.21068	6.35656

Int-1′

C	4.1657	0.0336	-2.26907
C	5.11649	-0.03134	-1.24918
H	2.06464	0.23238	-2.74557
C	2.81423	0.20001	-1.96303
C	4.7151	0.06561	0.08501
C	3.36653	0.23067	0.39797
C	2.4074	0.30338	-0.62407
H	5.45028	0.0066	0.88283

H	3.06258	0.29018	1.43914
C	0.46625	2.31413	0.2506
C	-0.69666	2.77582	0.89188
C	1.47327	3.23335	-0.08617
C	-0.83503	4.12682	1.20389
H	-1.49169	2.07713	1.12267
C	1.32312	4.58634	0.22024
H	2.37468	2.8968	-0.58687
C	0.1722	5.0347	0.86837
H	2.10882	5.28739	-0.04744
P	0.64391	0.55234	-0.22258
N	-0.08328	0.03843	-1.65943
N	-1.39545	0.01684	-1.73003
N	-2.04266	0.37301	-0.68194
H	-1.73757	4.47194	1.7006
H	0.05697	6.08858	1.10704
H	4.47378	-0.04989	-3.30767
H	6.16755	-0.1631	-1.49197
C	0.31978	-0.48078	1.25168
C	0.00527	-1.83242	1.04387
C	0.41625	0.01468	2.55951
C	-0.20847	-2.67498	2.13271
C	0.20215	-0.83546	3.64648
H	0.6465	1.06133	2.73341
C	-0.10979	-2.17877	3.43516
H	-0.45909	-3.71855	1.9642
H	0.27608	-0.4441	4.6575
C	-3.44386	0.34622	-0.81821
C	-4.12685	0.22398	-2.04526
C	-4.19194	0.47988	0.36504
C	-5.51375	0.22946	-2.07664
H	-3.55056	0.12773	-2.95819
C	-5.5795	0.48518	0.32929
H	-3.66083	0.56142	1.30913
C	-6.25846	0.36098	-0.89147
H	-6.0338	0.13702	-3.02396
H	-6.16154	0.58323	1.23995
C	-7.74091	0.37696	-0.87147
O	-8.42343	0.48911	0.13068
O	-8.28041	0.25102	-2.11149
C	-9.71249	0.25919	-2.15578
H	-10.10694	1.19725	-1.75508
H	-9.97374	0.1535	-3.20965
H	-10.12264	-0.57113	-1.57402

H	-0.08399	-2.21517	0.0312
H	-0.28007	-2.83739	4.28242

TS-2

C	-0.3627	3.93372	-3.18306
C	0.24389	4.8802	-2.34233
H	-1.06023	1.9312	-3.40552
C	-0.5634	2.6325	-2.74822
C	0.6518	4.49649	-1.06053
C	0.47194	3.17696	-0.64502
C	-0.1437	2.22176	-1.46742
H	1.12986	5.19954	-0.38868
H	0.82498	2.90416	0.34316
C	-1.90524	0.6017	0.30951
C	-2.52535	-0.58315	0.72414
C	-2.45576	1.82552	0.72709
C	-3.65315	-0.56256	1.5452
H	-2.13087	-1.53976	0.39489
C	-3.57837	1.85769	1.54319
H	-2.00931	2.76195	0.41003
C	-4.18625	0.66423	1.96101
H	-4.00856	2.80015	1.86725
P	-0.45493	0.51407	-0.81277
N	-1.84731	0.13615	-2.40597
N	-1.49294	-0.99112	-2.54254
N	-0.29949	-1.12515	-1.4278
H	-4.10737	-1.50058	1.84283
H	-0.67859	4.24645	-4.17339
C	1.00184	0.40796	0.40189
C	2.38478	0.40364	0.09069
C	0.69344	0.26944	1.76608
C	3.35541	0.21949	1.07934
C	1.66577	0.11189	2.75417
H	-0.34472	0.27408	2.07406
C	3.02019	0.06678	2.42839
H	4.39886	0.18671	0.78198
H	1.35425	0.01371	3.79147
C	0.55495	-2.22505	-1.30999
C	0.87926	-2.91582	-2.50094
C	1.03236	-2.74424	-0.08807
C	1.65179	-4.07004	-2.4702
H	0.48301	-2.5467	-3.44165
C	1.83163	-3.879	-0.06968

H	0.77585	-2.25303	0.84242
C	2.15088	-4.55817	-1.25399
H	1.8829	-4.59153	-3.39252
H	2.20841	-4.27234	0.86909
C	2.99923	-5.77222	-1.15937
O	3.44439	-6.22792	-0.12243
O	3.23456	-6.33651	-2.36991
C	4.05516	-7.51225	-2.34539
H	3.58778	-8.29657	-1.7436
H	4.14397	-7.82884	-3.38534
H	5.03995	-7.28652	-1.9272
C	4.09029	-0.14736	3.47048
H	3.6815	-0.07224	4.48273
H	4.54677	-1.13976	3.36732
H	4.89583	0.58913	3.37296
S	3.17065	0.76691	-1.5077
O	4.33977	-0.11281	-1.61008
O	3.33036	2.21944	-1.63294
N	2.02408	0.32966	-2.65383
H	1.99978	1.03223	-3.38936
H	2.18796	-0.61184	-3.0064
O	-5.27908	0.8043	2.7597
O	0.38568	6.12783	-2.86053
C	1.02049	7.12224	-2.06733
H	1.03655	8.02519	-2.67977
H	0.45869	7.31917	-1.14513
H	2.04847	6.83547	-1.81268
C	-5.95381	-0.36807	3.19426
H	-6.78816	-0.02171	3.80637
H	-6.34091	-0.94698	2.34597
H	-5.29967	-1.00784	3.80083

TS-2′

C	0.43584	3.5651	-2.38731
C	0.69471	4.44682	-1.33826
H	-0.27643	1.62143	-2.95415
C	-0.05252	2.28349	-2.12749
C	0.47922	4.03709	-0.02126
C	0.02213	2.74677	0.24448
C	-0.26257	1.85719	-0.80706
H	0.67861	4.71632	0.8032
H	-0.10939	2.43388	1.27498
C	-2.59156	0.19352	0.27414

C	-3.23797	-1.0214	0.54751
C	-3.27449	1.399	0.48422
C	-4.54253	-1.027	1.04062
H	-2.72847	-1.96349	0.36513
C	-4.57949	1.38665	0.97871
H	-2.79426	2.34594	0.25939
C	-5.215	0.17659	1.26058
H	-5.10008	2.32685	1.13953
P	-0.8947	0.15217	-0.42364
N	-1.77041	0.02578	-2.32525
N	-1.27052	-1.04269	-2.55494
N	-0.41471	-1.31401	-1.25437
H	-5.03379	-1.97371	1.24801
H	-6.2318	0.17069	1.64349
H	0.6039	3.87426	-3.41522
H	1.06412	5.44783	-1.54454
C	0.15631	-0.13602	1.10055
C	1.55357	-0.2004	0.95547
C	-0.38343	-0.24374	2.38998
C	2.38136	-0.38592	2.06012
C	0.44717	-0.42773	3.50039
H	-1.45692	-0.18846	2.53761
C	1.8293	-0.50226	3.33871
H	3.45803	-0.44146	1.92363
H	0.00777	-0.51165	4.49098
H	2.4743	-0.64759	4.20109
H	1.9993	-0.10915	-0.03159
C	0.22205	-2.53772	-0.98303
C	0.91423	-3.14652	-2.04945
C	0.13407	-3.22201	0.24496
C	1.49424	-4.39852	-1.89435
H	0.97716	-2.62455	-2.9982
C	0.74619	-4.45837	0.40245
H	-0.40137	-2.77813	1.0756
C	1.42748	-5.06533	-0.66177
H	2.01792	-4.85858	-2.72494
H	0.69141	-4.98378	1.35044
C	2.0467	-6.39403	-0.43044
O	2.01674	-7.0001	0.62457
O	2.66477	-6.87883	-1.53669
C	3.28297	-8.16242	-1.37608
H	2.54055	-8.91666	-1.10096
H	3.72647	-8.39705	-2.34457
H	4.05234	-8.12623	-0.59982

Int-2

C	0.14728	-0.74667	-3.80526
C	0.31733	-2.13678	-3.81635
H	0.07673	1.02281	-2.59813
C	0.24214	-0.04906	-2.60164
C	0.57575	-2.81658	-2.61437
C	0.66784	-2.11218	-1.42354
C	0.51876	-0.713	-1.40252
H	0.68393	-3.89616	-2.6417
H	0.84083	-2.66075	-0.50131
C	2.377	0.16102	0.69004
C	2.77914	1.04111	1.70606
C	3.34246	-0.69024	0.12352
C	4.09492	1.06836	2.16348
H	2.05657	1.72618	2.13993
C	4.65627	-0.67266	0.57115
H	3.07122	-1.3662	-0.68056
C	5.04331	0.20534	1.59582
H	5.40708	-1.32466	0.13605
P	0.63049	0.24873	0.14227
N	0.09376	1.75955	0.11692
H	4.37025	1.76575	2.94607
H	-0.0742	-0.20283	-4.71591
C	-0.34378	-0.68133	1.40857
C	-1.75277	-0.69649	1.54121
C	0.37875	-1.47018	2.31544
C	-2.37538	-1.45746	2.52504
C	-0.25146	-2.23668	3.29768
H	1.46108	-1.48838	2.26189
C	-1.63918	-2.24254	3.42374
H	-3.4592	-1.4339	2.57837
H	0.35601	-2.83374	3.97328
S	-2.90701	0.18377	0.44958
O	-2.45636	-0.03994	-0.93121
O	-4.27621	-0.19209	0.81977
N	-2.67855	1.75852	0.97588
H	-1.73745	2.05477	0.65765
C	-2.34055	-3.05065	4.48732
H	-2.79513	-2.39662	5.24195
H	-3.14775	-3.65497	4.05823
H	-1.64738	-3.72314	5.00158
C	0.71754	2.87252	-0.42812

C	1.83058	2.85343	-1.30506
C	0.19392	4.14441	-0.08479
C	2.3789	4.02812	-1.80338
H	2.26712	1.90433	-1.60069
C	0.741	5.3125	-0.58586
H	-0.6483	4.18819	0.60037
C	1.84464	5.27745	-1.45521
H	3.2307	3.98054	-2.47364
H	0.32853	6.27861	-0.31202
C	2.38741	6.55661	-1.95657
O	1.95596	7.66037	-1.67334
O	3.44961	6.38421	-2.79091
C	4.01527	7.59331	-3.3086
H	4.38127	8.22975	-2.49772
H	4.84048	7.28049	-3.95035
H	3.27361	8.15373	-3.88501
H	-3.42687	2.34064	0.59876
O	0.24411	-2.91659	-4.92677
O	6.34987	0.14612	1.96019
C	-0.06163	-2.29893	-6.17046
H	0.70481	-1.5666	-6.45467
H	-0.08087	-3.1053	-6.90531
H	-1.04203	-1.80725	-6.14308
C	6.81404	1.0277	2.97543
H	6.29719	0.85101	3.92721
H	7.87625	0.80913	3.09468
H	6.68861	2.07749	2.68239

Int-2′

P	-0.38023	-0.01783	-0.06715
C	0.05732	0.1075	1.70055
C	1.36813	0.46708	2.04403
C	-0.87639	-0.15774	2.71269
C	1.74077	0.54584	3.3854
H	2.07226	0.70176	1.25268
C	-0.4974	-0.07908	4.05385
H	-1.90392	-0.40444	2.45952
C	0.8114	0.26939	4.39099
H	2.75709	0.82865	3.64584
H	-1.22737	-0.28013	4.83325
H	1.10433	0.33395	5.43552
C	-2.16461	0.42033	-0.17325
C	-3.18549	-0.51829	-0.37862

C	-2.49279	1.78289	-0.07177
C	-4.51576	-0.10154	-0.46641
H	-2.94558	-1.57235	-0.4765
C	-3.82088	2.19431	-0.16119
H	-1.70402	2.51777	0.06405
C	-4.83438	1.25224	-0.35647
H	-5.30063	-0.83596	-0.62595
H	-4.06496	3.25023	-0.08472
H	-5.86951	1.57494	-0.42894
C	-0.25824	-1.80214	-0.50433
C	0.29849	-2.14417	-1.74579
C	-0.66145	-2.82357	0.37068
C	0.42704	-3.48433	-2.11349
H	0.63808	-1.36423	-2.41953
C	-0.53121	-4.16182	-0.00069
H	-1.06181	-2.57915	1.35028
C	0.00999	-4.49363	-1.24465
H	0.85928	-3.73778	-3.07751
H	-0.84393	-4.94454	0.68519
H	0.11436	-5.53665	-1.53117
N	0.63051	0.92819	-0.84069
C	0.62356	1.41726	-2.12995
C	-0.3657	1.15486	-3.11194
C	1.69643	2.26419	-2.50762
C	-0.28463	1.706	-4.38404
H	-1.20997	0.51647	-2.86603
C	1.77431	2.80844	-3.77688
H	2.45995	2.4746	-1.76473
C	0.78541	2.5408	-4.74054
H	-1.05702	1.48819	-5.11429
H	2.60221	3.45448	-4.0523
C	0.92207	3.14953	-6.07828
O	1.83212	3.88075	-6.42839
O	-0.09654	2.80845	-6.91707
C	-0.0134	3.37314	-8.22941
H	-0.89258	3.00811	-8.76302
H	-0.02014	4.46609	-8.18429
H	0.90159	3.05047	-8.73478

TS-3

C	1.12079	-0.56673	-3.8814
C	-0.18266	-0.94407	-4.24594
H	2.44815	-0.05834	-2.29329

C	1.43717	-0.3563	-2.54933
C	-1.16128	-1.1032	-3.25612
C	-0.83733	-0.88574	-1.91877
C	0.46277	-0.52092	-1.5418
H	-2.17723	-1.37936	-3.51188
H	-1.61788	-0.9665	-1.17328
C	2.50599	-0.89707	0.49774
C	3.29276	-0.35223	1.52199
C	2.99644	-2.01664	-0.20234
C	4.53625	-0.89296	1.84537
H	2.93148	0.51732	2.06302
C	4.23215	-2.5631	0.11061
H	2.41053	-2.45997	-1.00138
C	5.01294	-2.00572	1.13746
H	4.62103	-3.42106	-0.42836
P	0.86542	-0.16248	0.18981
N	0.90963	1.42993	0.60164
H	5.12108	-0.44282	2.63892
H	1.86293	-0.43837	-4.66259
C	-0.31364	-1.03766	1.30196
C	-1.57771	-0.57226	1.74732
C	0.08351	-2.32288	1.70643
C	-2.36851	-1.384	2.55601
C	-0.72661	-3.12509	2.50876
H	1.04579	-2.7152	1.39905
C	-1.96816	-2.668	2.9503
H	-3.32677	-0.98401	2.8736
H	-0.37738	-4.11422	2.79402
S	-2.27744	1.08088	1.34977
O	-2.25856	1.11289	-0.12771
O	-3.56938	1.12688	2.0573
N	-1.20929	2.14543	1.88682
H	-1.32607	2.2895	2.88945
C	1.42851	2.44742	-0.24409
C	2.75892	2.44506	-0.69889
C	0.59122	3.52386	-0.59057
C	3.23735	3.48379	-1.49166
H	3.42663	1.63941	-0.41167
C	1.07955	4.56457	-1.36915
H	-0.43764	3.5168	-0.24713
C	2.40358	4.55666	-1.83378
H	4.26535	3.47257	-1.83704
H	0.43805	5.39597	-1.64283
C	2.86284	5.6935	-2.67022

O	2.17723	6.64739	-2.98577
O	4.15773	5.56295	-3.06075
C	4.65786	6.6345	-3.87075
H	5.69291	6.37355	-4.09593
H	4.07619	6.72695	-4.79214
H	4.61071	7.58337	-3.32907
H	0.00129	1.79636	1.25738
C	-2.85935	-3.51182	3.82765
H	-3.8523	-3.63409	3.37937
H	-2.4358	-4.50716	3.99295
H	-3.00722	-3.04051	4.80704
O	-0.39011	-1.12831	-5.57359
O	6.20481	-2.61174	1.36223
C	-1.6996	-1.4652	-6.0177
H	-2.42464	-0.68737	-5.74907
H	-1.63304	-1.54073	-7.10403
H	-2.02729	-2.42777	-5.60525
C	7.04918	-2.09966	2.38725
H	7.93609	-2.73469	2.38449
H	7.3426	-1.06258	2.18256
H	6.564	-2.15516	3.36965

TS-3′

C	1.7589	2.62885	-2.00912
C	1.16041	3.87914	-1.84061
H	1.58332	0.50595	-1.69119
C	1.11975	1.47788	-1.54693
C	-0.08272	3.9744	-1.21209
C	-0.72818	2.82696	-0.74989
C	-0.12774	1.57132	-0.91222
H	-0.55685	4.94394	-1.08575
H	-1.70169	2.90782	-0.27664
C	-2.65841	0.32231	0.25677
C	-3.76901	-0.11574	-0.48104
C	-2.86411	0.96123	1.49174
C	-5.05639	0.07068	0.02175
H	-3.61078	-0.55582	-1.45634
C	-4.1574	1.17286	1.97198
H	-2.01956	1.2922	2.08718
C	-5.25586	0.71844	1.24235
H	-4.30101	1.68088	2.92176
P	-0.93001	0.04124	-0.31889
N	-0.58829	-1.33721	-1.18119

H	-5.90876	-0.28091	-0.55336
H	-6.26289	0.86988	1.62238
H	2.72309	2.54642	-2.50331
H	1.65853	4.77464	-2.20184
C	-0.11158	-0.26106	1.3232
C	0.87968	0.57852	1.84917
C	-0.51193	-1.38123	2.07267
C	1.46304	0.30173	3.08938
C	0.07196	-1.65897	3.30614
H	-1.28329	-2.04251	1.68763
C	1.06311	-0.81709	3.81803
H	2.23057	0.9636	3.48171
H	-0.24494	-2.53338	3.8679
H	1.51874	-1.03376	4.78049
H	1.20422	1.45285	1.29482
C	0.23565	-2.45347	-0.99091
C	-0.22066	-3.68569	-1.50248
C	1.51671	-2.40869	-0.40587
C	0.56557	-4.82718	-1.429
H	-1.20606	-3.72498	-1.95749
C	2.29313	-3.55685	-0.31916
H	1.90335	-1.47146	-0.02213
C	1.83294	-4.77947	-0.82739
H	0.19706	-5.76537	-1.82915
H	3.27906	-3.52218	0.13329
C	2.71491	-5.96471	-0.70897
O	3.81855	-5.96343	-0.19482
O	2.15871	-7.08281	-1.2426
C	2.966	-8.26387	-1.15743
H	3.18555	-8.50819	-0.11431
H	2.37609	-9.05618	-1.62025
H	3.91	-8.12654	-1.69227
O	-1.89074	0.1053	-2.46393
H	-1.15734	-1.01725	-2.12023
H	-1.35775	0.69213	-3.02237

Int-3

C	0.40183	-1.34706	-3.63674
C	-0.88684	-1.75855	-4.01684
H	1.69349	-0.79504	-2.03494
C	0.69277	-1.11591	-2.30248
C	-1.87624	-1.93169	-3.03998
C	-1.57909	-1.69139	-1.70081

C	-0.29424	-1.28996	-1.30862
H	-2.8811	-2.23537	-3.30788
H	-2.36791	-1.78104	-0.96585
C	1.72815	-1.65033	0.74587
C	2.51038	-1.0987	1.77024
C	2.22038	-2.77551	0.05569
C	3.75296	-1.63678	2.10073
H	2.14625	-0.22713	2.30558
C	3.45466	-3.31964	0.37694
H	1.63691	-3.22451	-0.74182
C	4.2322	-2.75456	1.40229
H	3.84501	-4.18179	-0.15414
P	0.08394	-0.93612	0.42754
N	0.16081	0.67082	0.84913
H	4.33478	-1.18121	2.89332
H	1.15313	-1.21035	-4.40763
C	-1.09717	-1.79229	1.54364
C	-2.37916	-1.32898	1.93671
C	-0.68363	-3.0535	2.00483
C	-3.17546	-2.12777	2.75314
C	-1.5	-3.83882	2.8169
H	0.29276	-3.43887	1.73535
C	-2.7622	-3.38833	3.20546
H	-4.15096	-1.73642	3.02644
H	-1.14093	-4.80901	3.15069
S	-3.07363	0.31494	1.47972
O	-2.90009	0.33372	0.00758
O	-4.44188	0.30293	2.0343
N	-2.0955	1.3926	2.11424
H	-2.35096	1.55977	3.08763
C	0.65859	1.68982	-0.01304
C	1.97705	1.6766	-0.49575
C	-0.18624	2.7639	-0.34112
C	2.43878	2.70861	-1.30732
H	2.64857	0.87169	-0.21552
C	0.28768	3.79864	-1.13701
H	-1.20579	2.7593	0.02992
C	1.59939	3.78149	-1.63483
H	3.45815	2.69298	-1.6769
H	-0.35672	4.63143	-1.3991
C	2.04184	4.91164	-2.49081
O	1.35234	5.86692	-2.79227
O	3.32403	4.771	-2.91626
C	3.80762	5.83431	-3.74748

H	4.83463	5.56593	-3.99895
H	3.2012	5.92311	-4.65308
H	3.7802	6.78703	-3.21133
H	-0.68591	1.00768	1.44374
C	-3.66654	-4.22145	4.07971
H	-4.58539	-4.49422	3.54629
H	-3.17651	-5.14532	4.40179
H	-3.97051	-3.66669	4.97507
O	-1.06951	-1.96135	-5.34487
O	5.42293	-3.35853	1.63524
C	-2.36372	-2.33484	-5.80532
H	-3.1112	-1.57207	-5.55603
H	-2.27834	-2.42001	-6.88958
H	-2.67401	-3.30073	-5.38738
C	6.26368	-2.84038	2.66059
H	5.77449	-2.88978	3.64125
H	7.15022	-3.47584	2.66505
H	6.55823	-1.80476	2.45045

Int-3′

C	2.01817	3.74685	-1.81697
C	1.50034	5.02159	-1.58609
H	1.6901	1.62717	-1.65519
C	1.27525	2.61489	-1.47301
C	0.23036	5.16006	-1.02126
C	-0.51994	4.03164	-0.68962
C	0.0016	2.74759	-0.9021
H	-0.18252	6.14995	-0.8453
H	-1.51418	4.15027	-0.26935
C	-2.63446	1.49334	0.33392
C	-3.82518	0.98012	-0.20449
C	-2.69414	2.19629	1.54687
C	-5.03724	1.14928	0.46453
H	-3.80191	0.4632	-1.15574
C	-3.91448	2.40141	2.19288
H	-1.78631	2.58335	1.99765
C	-5.08794	1.86773	1.66025
H	-3.94164	2.96565	3.12135
P	-1.00028	1.23611	-0.53288
N	-0.50883	-0.24076	-1.30638
H	-5.94627	0.73005	0.04103
H	-6.03558	2.01046	2.17304
H	3.00299	3.62889	-2.26138

H	2.08034	5.90232	-1.84822
C	-0.12305	0.78299	1.12326
C	0.88367	1.55125	1.72528
C	-0.57705	-0.35027	1.82268
C	1.42896	1.19323	2.96438
C	-0.03598	-0.71601	3.0528
H	-1.36986	-0.96225	1.39828
C	0.97502	0.05719	3.63003
H	2.20998	1.80838	3.40489
H	-0.40291	-1.60321	3.56283
H	1.3995	-0.22342	4.59049
H	1.25855	2.44288	1.23396
C	0.34197	-1.33074	-1.03056
C	-0.08566	-2.609	-1.43811
C	1.62421	-1.20181	-0.46884
C	0.73223	-3.72092	-1.28891
H	-1.07637	-2.7217	-1.87254
C	2.43231	-2.31916	-0.30478
H	1.98377	-0.22971	-0.15668
C	2.00337	-3.59012	-0.71175
H	0.38487	-4.69655	-1.61017
H	3.42125	-2.22112	0.131
C	2.92196	-4.73863	-0.51819
O	4.03217	-4.66548	-0.02546
O	2.38975	-5.90825	-0.95546
C	3.23078	-7.05806	-0.79348
H	3.46786	-7.21757	0.26204
H	2.65782	-7.898	-1.18846
H	4.16446	-6.93508	-1.34927
O	-1.9176	1.52784	-2.04315
H	-1.14264	-0.41697	-2.07659
H	-1.30051	1.76066	-2.75528

Int-4

C	-3.0231	-0.85277	-3.64129
C	-2.25326	-1.24955	-4.74248
H	-3.02606	-0.38737	-1.54936
C	-2.4141	-0.68454	-2.39678
C	-0.87611	-1.46305	-4.58203
C	-0.27703	-1.27519	-3.34182
C	-1.04027	-0.88793	-2.22873
H	-0.29451	-1.77192	-5.445
H	0.79082	-1.44351	-3.24383

C	1.38773	-1.30562	-0.2486
C	1.6415	-2.14852	0.84321
C	2.4766	-0.96294	-1.07672
C	2.92013	-2.63879	1.10847
H	0.84246	-2.41972	1.5234
C	3.74357	-1.47623	-0.84881
H	2.32707	-0.27082	-1.89651
C	3.97937	-2.31584	0.25143
H	4.57693	-1.22128	-1.49575
P	-0.30469	-0.63089	-0.55084
N	0.35551	0.99546	-1.16599
H	3.07383	-3.26689	1.97822
H	-4.08852	-0.67826	-3.73658
C	-1.2148	-2.20363	0.13305
C	-2.01737	-2.0703	1.25954
C	-1.16993	-3.47925	-0.44845
C	-2.76366	-3.10811	1.82082
C	-1.89431	-4.53678	0.09482
H	-0.56731	-3.64777	-1.33755
C	-2.7062	-4.37241	1.23284
H	-3.38432	-2.9299	2.69464
H	-1.83885	-5.51655	-0.37517
S	-1.98798	-0.44369	1.96688
O	-3.33781	0.11298	2.09914
O	-1.09104	-0.39244	3.1297
N	-1.22312	0.26528	0.61419
C	1.03011	2.02582	-0.53038
C	1.20352	3.2605	-1.20353
C	1.56622	1.90938	0.77423
C	1.88035	4.31463	-0.61421
H	0.79656	3.37458	-2.20648
C	2.2419	2.97383	1.35607
H	1.46166	0.98508	1.33053
C	2.41432	4.18919	0.67979
H	1.99891	5.24799	-1.1539
H	2.64958	2.87695	2.3573
C	3.14596	5.27842	1.36051
O	3.62994	5.2068	2.47581
O	3.23123	6.40343	0.59997
C	3.92833	7.49598	1.20992
H	4.96294	7.2201	1.4332
H	3.89833	8.30708	0.48079
H	3.4379	7.79807	2.13958
H	-0.97809	1.2396	0.77311

H	-0.07119	1.28472	-2.03884
C	-3.50216	-5.53017	1.78729
H	-2.86384	-6.40351	1.9663
H	-3.98516	-5.26678	2.73293
H	-4.28771	-5.8431	1.0879
O	-2.74121	-1.45292	-5.99671
O	5.25656	-2.7512	0.4001
C	-4.12983	-1.25387	-6.22272
H	-4.42675	-0.21754	-6.01616
H	-4.29362	-1.47257	-7.27914
H	-4.7383	-1.93348	-5.61225
C	5.5679	-3.57251	1.51861
H	6.63474	-3.78752	1.44106
H	5.36857	-3.05338	2.46436
H	5.00405	-4.5137	1.49423

Int-4′

C	0.87302	4.53244	-1.16919
C	0.06154	4.975	-2.21371
H	1.24495	2.98583	0.27521
C	0.601	3.31853	-0.53266
C	-1.03556	4.20752	-2.61108
C	-1.32554	3.00906	-1.96009
C	-0.50418	2.54949	-0.92062
H	-1.67367	4.5464	-3.42266
H	-2.19748	2.43219	-2.25533
C	-2.61687	1.58954	0.50028
C	-2.5973	2.72718	1.32675
C	-3.86755	1.03275	0.1922
C	-3.77136	3.28716	1.82654
H	-1.64823	3.18991	1.58876
C	-5.0498	1.59231	0.68976
H	-3.92098	0.15478	-0.44017
C	-5.0075	2.71963	1.50757
H	-6.00516	1.1403	0.4338
P	-0.93359	0.91948	-0.13983
N	0.66757	0.26508	-0.97756
H	-3.7221	4.16688	2.46351
H	-5.9263	3.15379	1.8935
H	1.72343	5.12785	-0.84776
H	0.2797	5.91481	-2.71347
C	-0.26924	0.51776	1.54719
C	1.08916	0.65934	1.87227

C	-1.14404	0.01967	2.5266
C	1.55174	0.35031	3.15118
C	-0.67238	-0.3291	3.79205
H	-2.19776	-0.0997	2.3011
C	0.67366	-0.15054	4.11292
H	2.604	0.48253	3.38747
H	-1.36209	-0.73132	4.529
H	1.03806	-0.40783	5.1039
H	1.79184	0.98579	1.11549
C	1.28941	-0.96837	-0.98733
C	0.76224	-2.09983	-0.31701
C	2.50317	-1.14117	-1.70177
C	1.41668	-3.3236	-0.36339
H	-0.15746	-2.01506	0.25115
C	3.14475	-2.36521	-1.74348
H	2.93017	-0.28841	-2.22636
C	2.61518	-3.48249	-1.07479
H	0.99316	-4.17378	0.16062
H	4.07224	-2.48364	-2.29507
C	3.34639	-4.76222	-1.15421
O	4.39003	-4.93677	-1.75903
O	2.72848	-5.76176	-0.46699
C	3.39561	-7.02801	-0.50798
H	2.7771	-7.70509	0.08327
H	3.48188	-7.38887	-1.53693
H	4.39874	-6.95283	-0.07843
O	-1.75622	-0.23405	-0.99601
H	-1.11896	-0.71139	-1.55538
H	1.14189	0.95221	-1.5515

TS-4

C	-2.18884	0.20775	-3.79405
C	-1.33454	-0.2354	-4.81288
H	-2.43705	0.52141	-1.68925
C	-1.75739	0.1881	-2.46821
C	-0.05141	-0.704	-4.48676
C	0.37361	-0.72134	-3.16636
C	-0.47207	-0.26392	-2.13944
H	0.59485	-1.04748	-5.28815
H	1.37049	-1.08601	-2.93948
C	1.78063	-0.39955	-0.11604
C	2.28408	-1.12279	0.9753
C	2.68367	0.30709	-0.93469

C	3.65093	-1.16154	1.24292
H	1.6098	-1.66046	1.63428
C	4.04451	0.25003	-0.69317
H	2.31148	0.9189	-1.74703
C	4.54101	-0.47873	0.40163
H	4.74688	0.79323	-1.31635
P	0.00012	-0.33692	-0.392
N	0.15548	2.20254	-0.63775
H	4.00723	-1.71952	2.10072
H	-3.18711	0.56592	-4.0163
C	-0.60943	-2.00242	0.14251
C	-1.71947	-1.97012	0.98581
C	-0.12014	-3.25181	-0.25235
C	-2.34218	-3.12269	1.45497
C	-0.73555	-4.41469	0.20668
H	0.74183	-3.32032	-0.91025
C	-1.84744	-4.37275	1.06598
H	-3.19912	-3.05022	2.11884
H	-0.3459	-5.38036	-0.1064
S	-2.31027	-0.32316	1.39319
O	-3.54181	-0.03681	0.64407
O	-2.30724	-0.12061	2.84179
N	-1.00411	0.47761	0.67935
C	1.17206	3.04567	-0.27559
C	1.72326	4.01698	-1.15716
C	1.73841	2.97168	1.02804
C	2.75857	4.85054	-0.76715
H	1.30797	4.10589	-2.1601
C	2.77666	3.80049	1.40784
H	1.33092	2.25162	1.73237
C	3.31261	4.75564	0.52221
H	3.15268	5.58435	-1.46285
H	3.19789	3.73406	2.4065
C	4.42062	5.60303	0.98882
O	4.94561	5.54111	2.08891
O	4.83119	6.50162	0.04526
C	5.90468	7.35377	0.45112
H	6.79528	6.76951	0.70197
H	6.10448	8.00354	-0.40297
H	5.62267	7.94901	1.32477
H	-0.88745	1.52652	0.39524
H	-0.17396	2.42472	-1.57751
C	-2.4769	-5.64781	1.5734
H	-1.88717	-6.07672	2.39386

H	-3.48822	-5.47222	1.95162
H	-2.53407	-6.40523	0.78426
O	5.88531	-0.45575	0.55923
O	-1.65019	-0.2559	-6.1332
C	-2.93164	0.21549	-6.53327
H	-3.07387	1.26857	-6.26027
H	-2.9579	0.1159	-7.61934
H	-3.73594	-0.38696	-6.09274
C	6.45312	-1.09806	1.69536
H	7.52659	-0.91539	1.63207
H	6.06476	-0.67223	2.62839
H	6.26618	-2.17932	1.67921

TS-4′

C	1.29408	3.84357	-1.72891
C	0.41667	4.78765	-2.2645
H	1.49938	1.92949	-0.79047
C	0.80049	2.67466	-1.14959
C	-0.95876	4.56265	-2.20666
C	-1.4565	3.41281	-1.59345
C	-0.58274	2.45255	-1.05821
H	-1.65019	5.28594	-2.62976
H	-2.52905	3.26457	-1.53004
C	-2.73596	1.8144	0.60134
C	-2.41489	2.72743	1.61993
C	-4.08938	1.59575	0.30677
C	-3.4114	3.40418	2.32013
H	-1.37374	2.91238	1.87501
C	-5.0911	2.27276	1.01006
H	-4.35884	0.89332	-0.47343
C	-4.75638	3.17775	2.01591
H	-6.13465	2.08829	0.7677
P	-1.33762	0.94089	-0.30711
N	0.0522	-0.17959	-1.59795
H	-3.13963	4.10592	3.1044
H	-5.53579	3.70289	2.56183
H	2.36726	4.00675	-1.77374
H	0.80377	5.69056	-2.72868
C	-0.57288	0.17984	1.1784
C	0.77146	0.31892	1.54169
C	-1.42052	-0.60125	1.98039
C	1.25529	-0.2967	2.69643
C	-0.92619	-1.23992	3.11628

H	-2.46867	-0.70838	1.71816
C	0.41187	-1.08254	3.48129
H	2.29935	-0.17394	2.96982
H	-1.59035	-1.85218	3.71996
H	0.79588	-1.57266	4.37165
H	1.45317	0.89799	0.93234
C	0.94305	-1.20798	-1.3605
C	0.58119	-2.33643	-0.5825
C	2.25169	-1.19811	-1.91061
C	1.47334	-3.37218	-0.35839
H	-0.41983	-2.39046	-0.1665
C	3.1356	-2.24127	-1.69309
H	2.55693	-0.35106	-2.52308
C	2.76821	-3.34583	-0.90674
H	1.16758	-4.2241	0.23974
H	4.13244	-2.22137	-2.12306
C	3.75455	-4.42229	-0.70264
O	4.88612	-4.43689	-1.15711
O	3.27208	-5.43737	0.06924
C	4.18771	-6.51357	0.29386
H	3.65021	-7.23272	0.91431
H	4.48732	-6.97406	-0.6521
H	5.08575	-6.16035	0.80929
O	-2.34794	0.03772	-1.2078
H	-1.66891	-0.44308	-1.77078
H	0.44273	0.46939	-2.27731

Product

C	-1.98758	0.38623	-3.83128
C	-1.19009	-0.15839	-4.84648
H	-2.23267	0.64371	-1.70825
C	-1.61124	0.23028	-2.4975
C	-0.0155	-0.8571	-4.51491
C	0.35172	-1.00808	-3.18706
C	-0.44164	-0.46305	-2.15938
H	0.58757	-1.26757	-5.31852
H	1.26664	-1.54421	-2.94972
C	1.70718	-0.58274	-0.17844
C	2.33753	-1.30126	0.8465
C	2.47607	0.31378	-0.94479
C	3.69958	-1.15158	1.09956
H	1.76262	-1.98585	1.46282
C	3.83109	0.47236	-0.70017

H	2.00961	0.89229	-1.73603
C	4.45418	-0.2594	0.32433
H	4.43207	1.16204	-1.28397
P	-0.08449	-0.69859	-0.39719
H	4.15709	-1.72424	1.89774
H	-2.89591	0.92944	-4.06343
C	-0.6808	-2.33687	0.14817
C	-1.73816	-2.16417	1.04029
C	-0.27815	-3.62704	-0.20439
C	-2.40288	-3.24856	1.59838
C	-0.93932	-4.71879	0.35799
H	0.53927	-3.78692	-0.90261
C	-2.00096	-4.54926	1.26373
H	-3.22317	-3.08554	2.29229
H	-0.62866	-5.72553	0.0895
S	-2.17193	-0.41743	1.33962
O	-3.45745	-0.17415	0.6602
O	-2.10871	-0.16236	2.78329
N	-0.92866	0.29746	0.54809
C	-2.68171	-5.74521	1.88608
H	-2.21418	-6.0072	2.84439
H	-3.73883	-5.54293	2.08548
H	-2.61777	-6.62589	1.23897
O	5.78245	-0.03326	0.48274
O	-1.45985	-0.06666	-6.174
C	-2.6375	0.61946	-6.58248
H	-2.6165	1.67142	-6.27155
H	-2.64977	0.56368	-7.67203
H	-3.53931	0.13966	-6.18237
C	6.47033	-0.71226	1.52661
H	7.5029	-0.36387	1.47616
H	6.04964	-0.46532	2.50919
H	6.4459	-1.79976	1.38184

Ph_3PO

P	0.002	0.0029	0.91568
C	-1.18149	-1.20814	0.21609
C	-1.49804	-2.31501	1.01763
C	-1.77446	-1.08191	-1.04842
C	-2.37935	-3.29049	0.55161
H	-1.06184	-2.39055	2.00928
C	-2.65408	-2.06121	-1.51282
H	-1.56745	-0.21175	-1.66541

C	-2.95431	-3.16716	-0.71511
H	-2.62174	-4.14387	1.17929
H	-3.11197	-1.95516	-2.49275
H	-3.64266	-3.92661	-1.07659
C	-0.45666	1.63259	0.21591
C	-0.04162	2.08826	-1.04368
C	-1.26851	2.45408	1.0123
C	-0.45218	3.33887	-1.50844
H	0.61706	1.47871	-1.65623
C	-1.67482	3.70414	0.54595
H	-1.55964	2.11048	2.00053
C	-1.27118	4.14561	-0.71603
H	-0.12438	3.68663	-2.48454
H	-2.30151	4.33606	1.16957
H	-1.58631	5.12077	-1.078
C	1.64144	-0.41782	0.21475
C	2.75967	-0.13781	1.01415
C	1.82587	-0.99973	-1.04771
C	4.04391	-0.41867	0.54803
H	2.60905	0.2816	2.0045
C	3.11261	-1.27708	-1.5123
H	0.96695	-1.2557	-1.66196
C	4.22189	-0.98389	-0.71662
H	4.9054	-0.20189	1.174
H	3.24769	-1.73104	-2.49047
H	5.22286	-1.20456	-1.07801
O	0.00311	0.00255	2.4192

N_2

N	1.85312	-1.98749	7.03701
N	1.19754	-2.09222	6.15306

4-CO_2Me-PhNH_2

C	0.24976	0.06222	1.72716
C	1.1781	0.39425	2.72632
C	-0.60066	-1.03466	1.93564
C	1.25335	-0.34534	3.89794
H	1.84007	1.24053	2.57942
C	-0.53117	-1.77878	3.10289
H	-1.31831	-1.28725	1.1617
C	0.39968	-1.44601	4.10692
H	1.97945	-0.07955	4.66294

H	-1.19387	-2.62885	3.24858
C	0.1227	0.81825	0.46153
O	-0.67423	0.56675	-0.42419
O	1.00481	1.84876	0.37973
C	0.9289	2.61939	-0.8262
H	-0.05979	3.07451	-0.93327
H	1.69588	3.38928	-0.73093
H	1.12269	1.99098	-1.69995
N	0.50951	-2.21944	5.25436
H	-0.30381	-2.76779	5.50161
H	0.935	-1.77161	6.05539

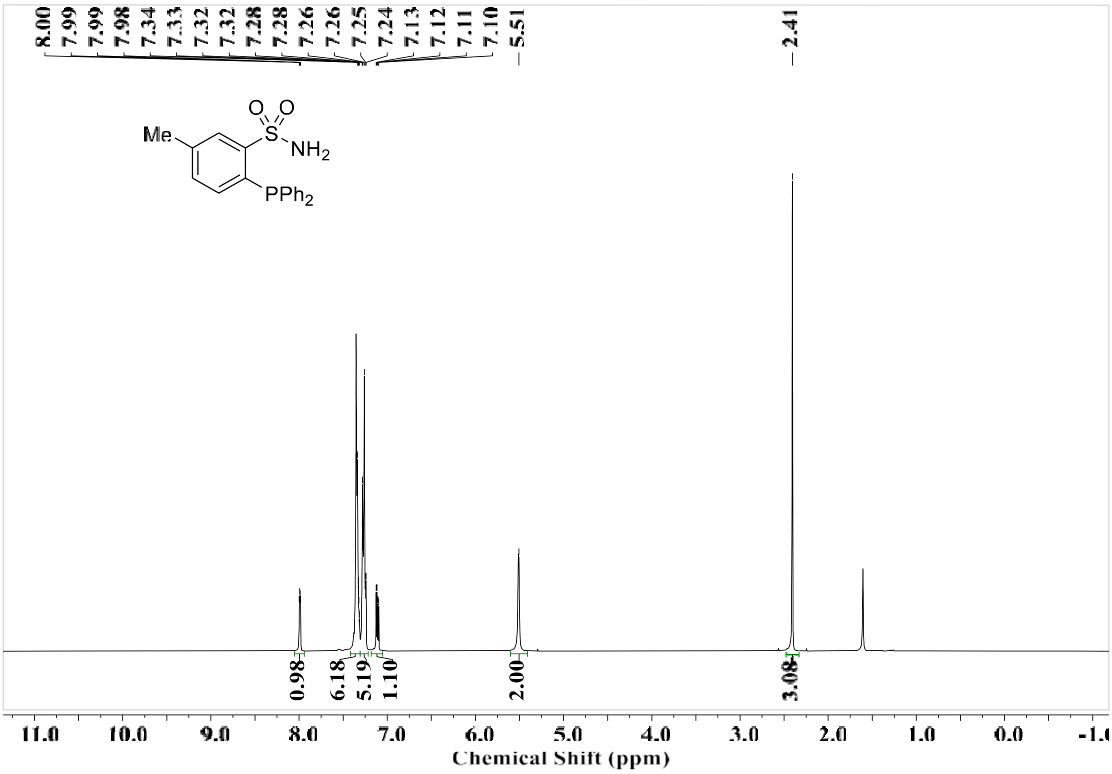
8.00
7.99
7.99
7.98
7.34
7.33
7.32
7.32
7.28
7.28
7.26
7.26
7.25
7.24
7.13
7.12
7.11
7.10
5.51
2.41
Me
O O
S
NH2
PPh2
0.98
6.18
5.19
1.10
2.00
3.08
11.0 10.0 9.0 8.0 7.0 6.0 5.0 4.0 3.0 2.0 1.0 0.0 -1.0
Chemical Shift (ppm)

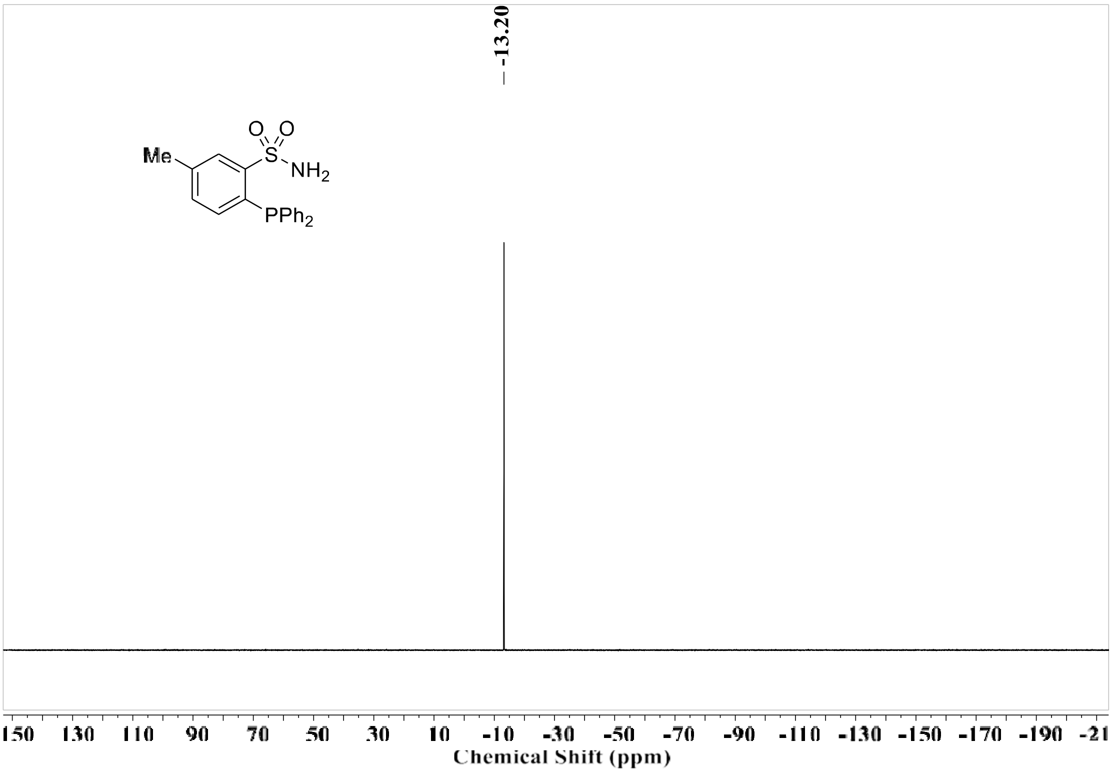
-13.20
Me
O O
S
NH2
PPh2
150 130 110 90 70 50 30 10 -10 -30 -50 -70 -90 -110 -130 -150 -170 -190 -21
Chemical Shift (ppm)

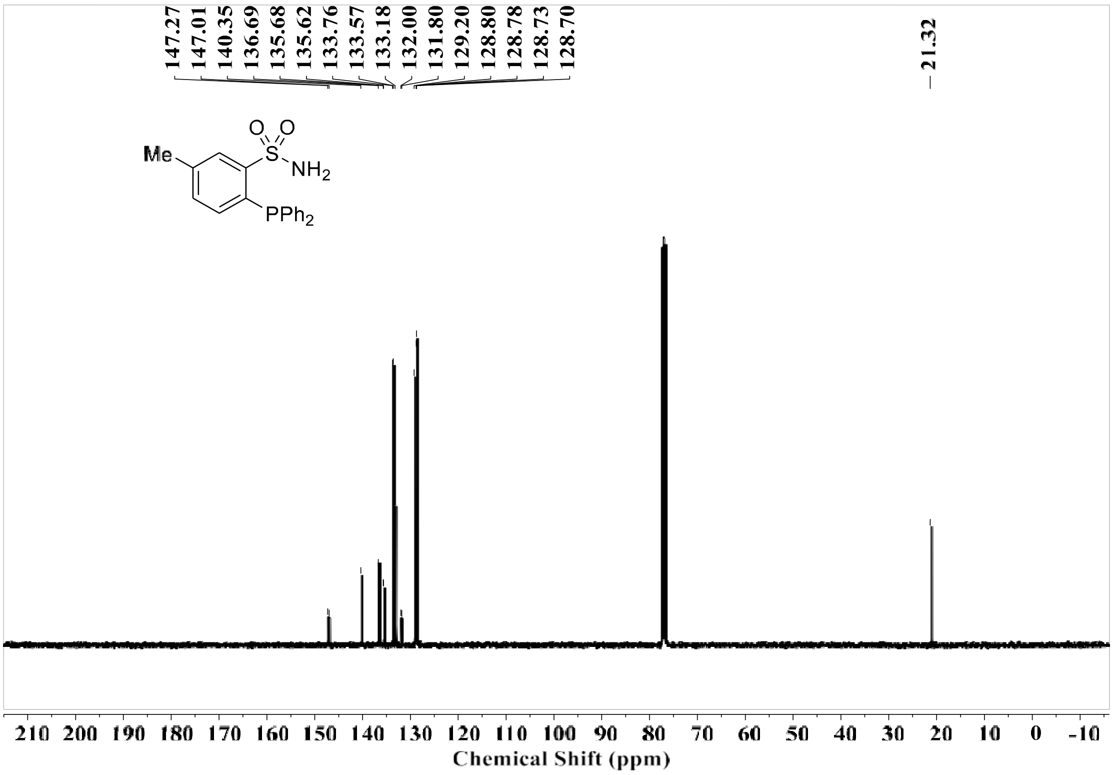

147.27
147.01
140.35
136.69
135.68
135.62
133.76
133.57
133.18
132.00
131.80
129.20
128.80
128.78
128.73
128.70
21.32
Me
O O
S
NH2
PPh2
210 200 190 180 170 160 150 140 130 120 110 100 90 80 70 60 50 40 30 20 10 0 -10
Chemical Shift (ppm)

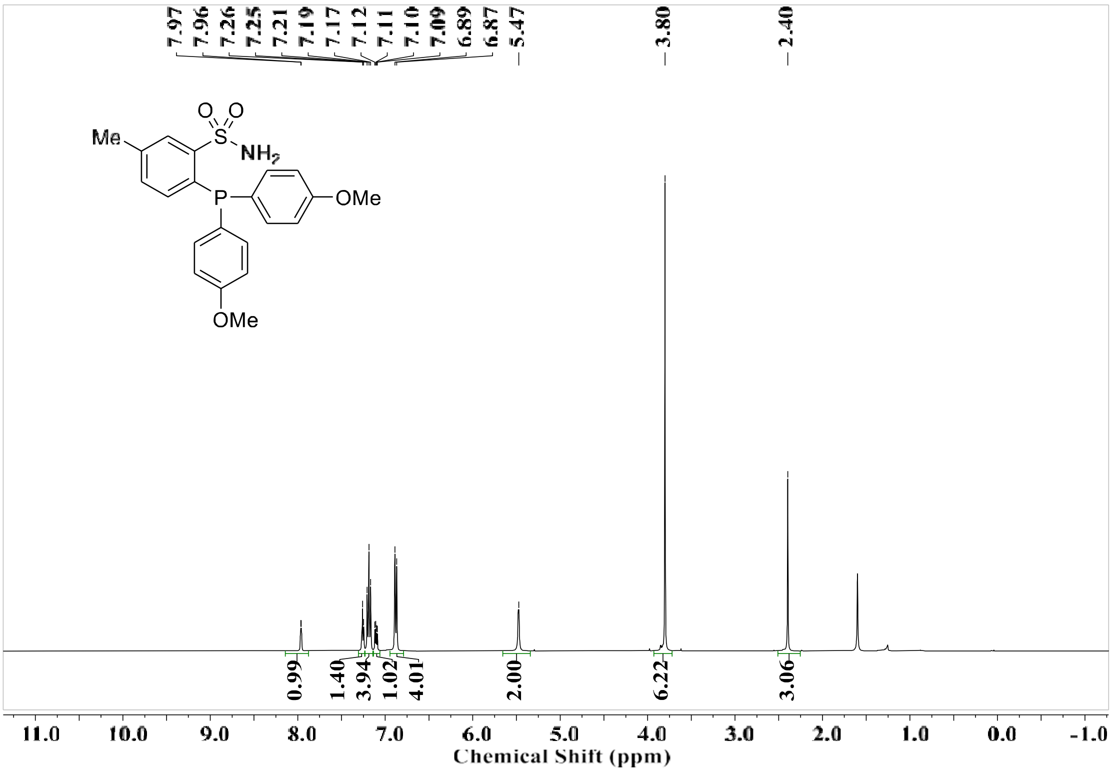

7.97
7.96
7.26
7.25
7.21
7.19
7.17
7.12
7.11
7.10
7.09
6.89
6.87
5.47
3.80
2.40
Me
O O
S
NH2
P
OMe
OMe
0.99
1.40
3.94
1.02
4.01
2.00
6.22
3.06
11.0 10.0 9.0 8.0 7.0 6.0 5.0 4.0 3.0 2.0 1.0 0.0 -1.0
Chemical Shift (ppm)

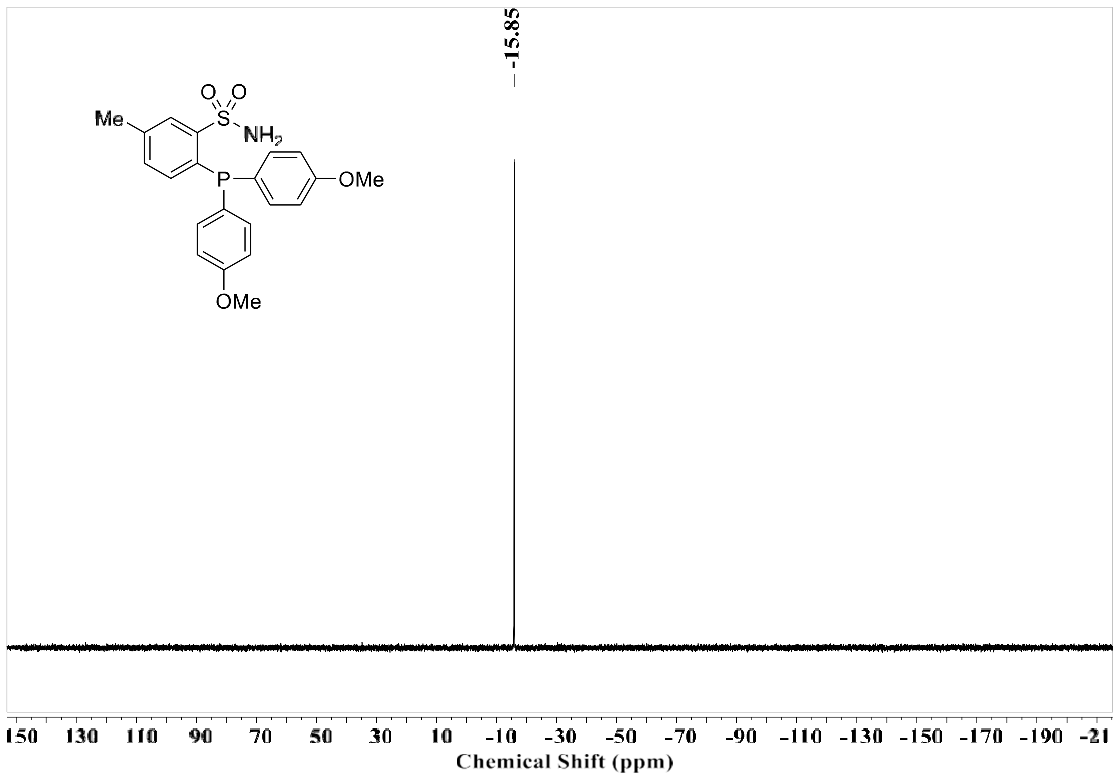
-15.85
Me
O O
S
NH2
P
OMe
OMe
150 130 110 90 70 50 30 10 -10 -30 -50 -70 -90 -110 -130 -150 -170 -190 -21
Chemical Shift (ppm)

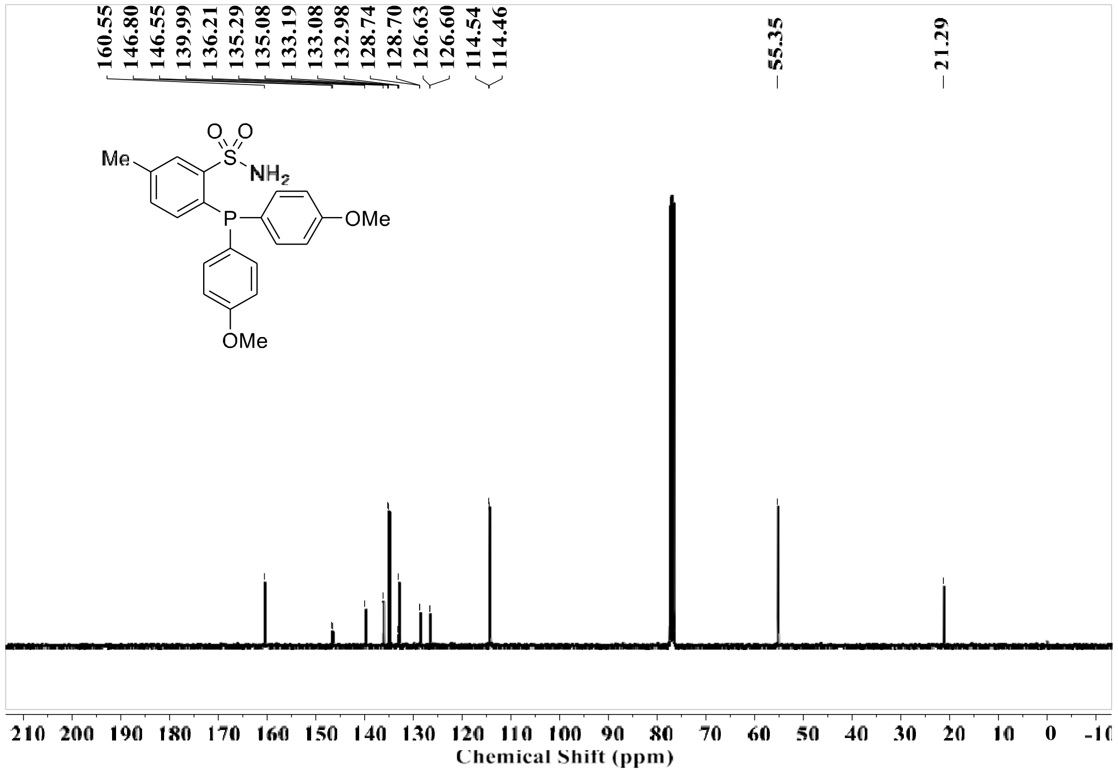
160.55
146.80
146.55
139.99
136.21
135.29
135.08
133.19
133.08
132.98
128.74
128.70
126.63
126.60
114.54
114.46
55.35
21.29
Me
O O
S
NH2
P
OMe
OMe
210 200 190 180 170 160 150 140 130 120 110 100 90 80 70 60 50 40 30 20 10 0 -10
Chemical Shift (ppm)

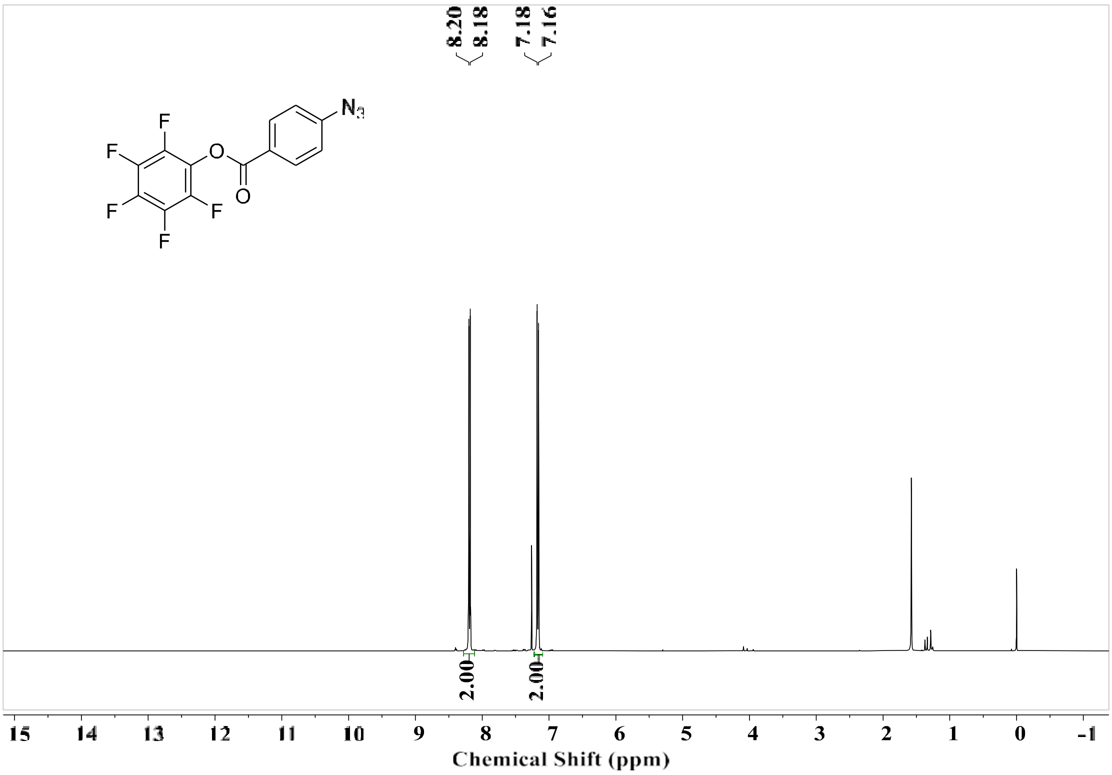

8.20
8.18
7.18
7.16
F
F
F
F
F
O
O
N3
2.00
2.00
15 14 13 12 11 10 9 8 7 6 5 4 3 2 1 0 -1
Chemical Shift (ppm)

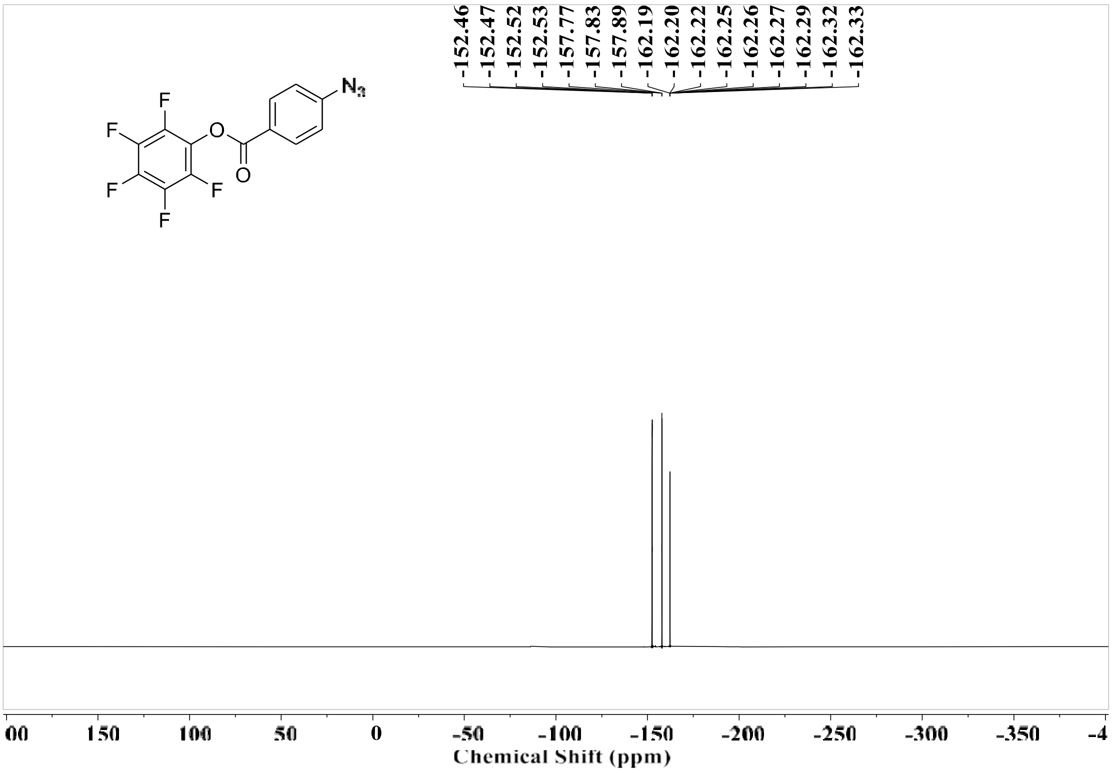

-152.46
-152.47
-152.52
-152.53
-157.77
-157.83
-157.89
-162.19
-162.20
-162.22
-162.25
-162.26
-162.27
-162.29
-162.32
-162.33
F
F
F
F
F
O
O
N3
00 150 100 50 0 -50 -100 -150 -200 -250 -300 -350 -4
Chemical Shift (ppm)

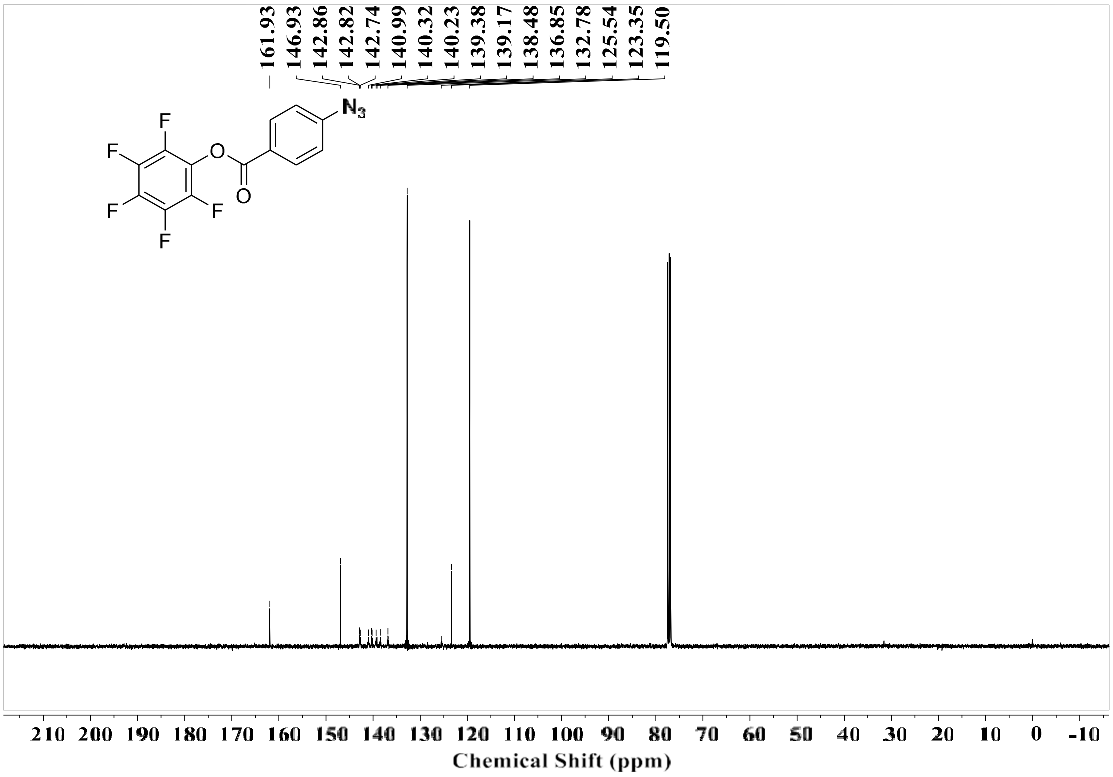
161.93
146.93
142.86
142.82
142.74
140.99
140.32
140.23
139.38
139.17
138.48
136.85
132.78
125.54
123.35
119.50
210 200 190 180 170 160 150 140 130 120 110 100 90 80 70 60 50 40 30 20 10 0 -10
Chemical Shift (ppm)

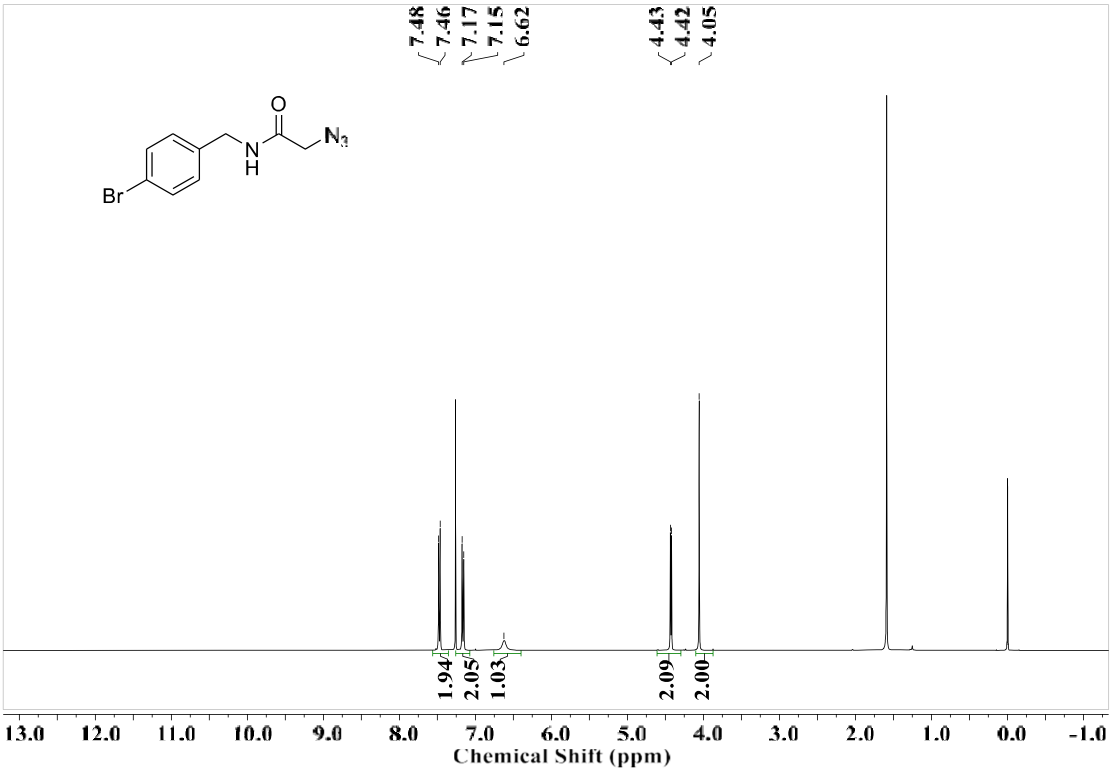
7.48
7.46
7.17
7.15
6.62
4.43
4.42
4.05
1.94
2.05
1.03
2.09
2.00
13.0 12.0 11.0 10.0 9.0 8.0 7.0 6.0 5.0 4.0 3.0 2.0 1.0 0.0 -1.0
Chemical Shift (ppm)

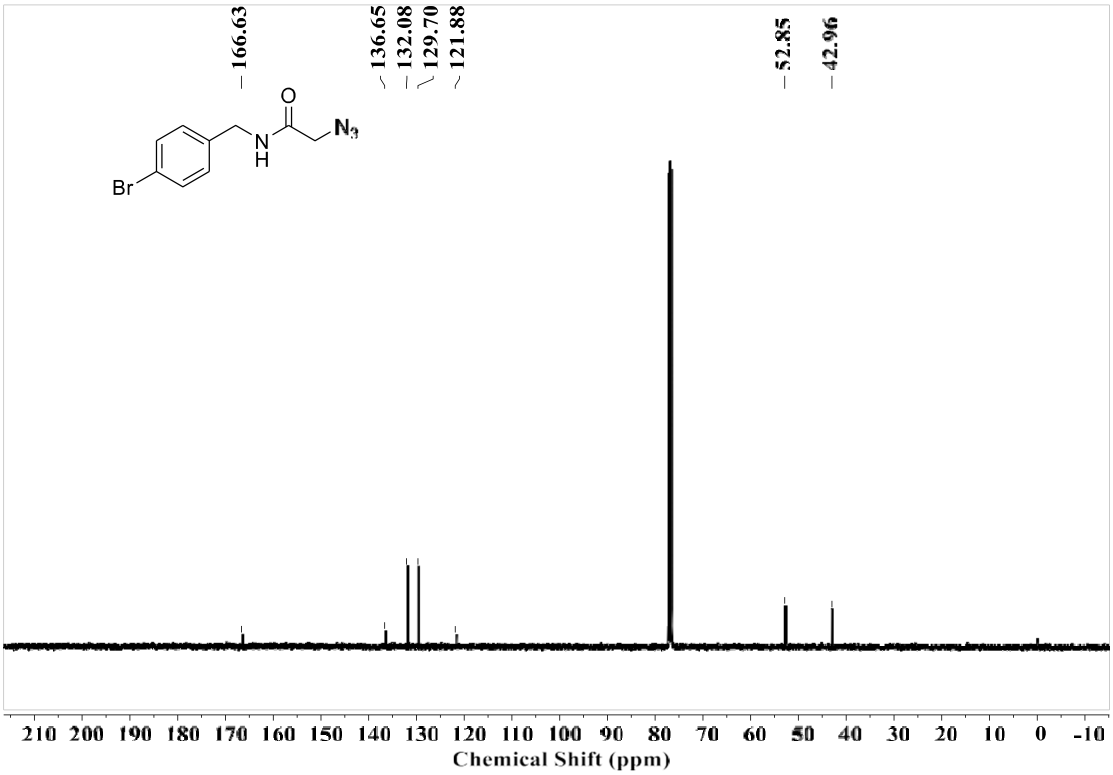

166.63
136.65
132.08
129.70
121.88
52.85
42.96
Br
N
H
O
N3
210 200 190 180 170 160 150 140 130 120 110 100 90 80 70 60 50 40 30 20 10 0 -10
Chemical Shift (ppm)

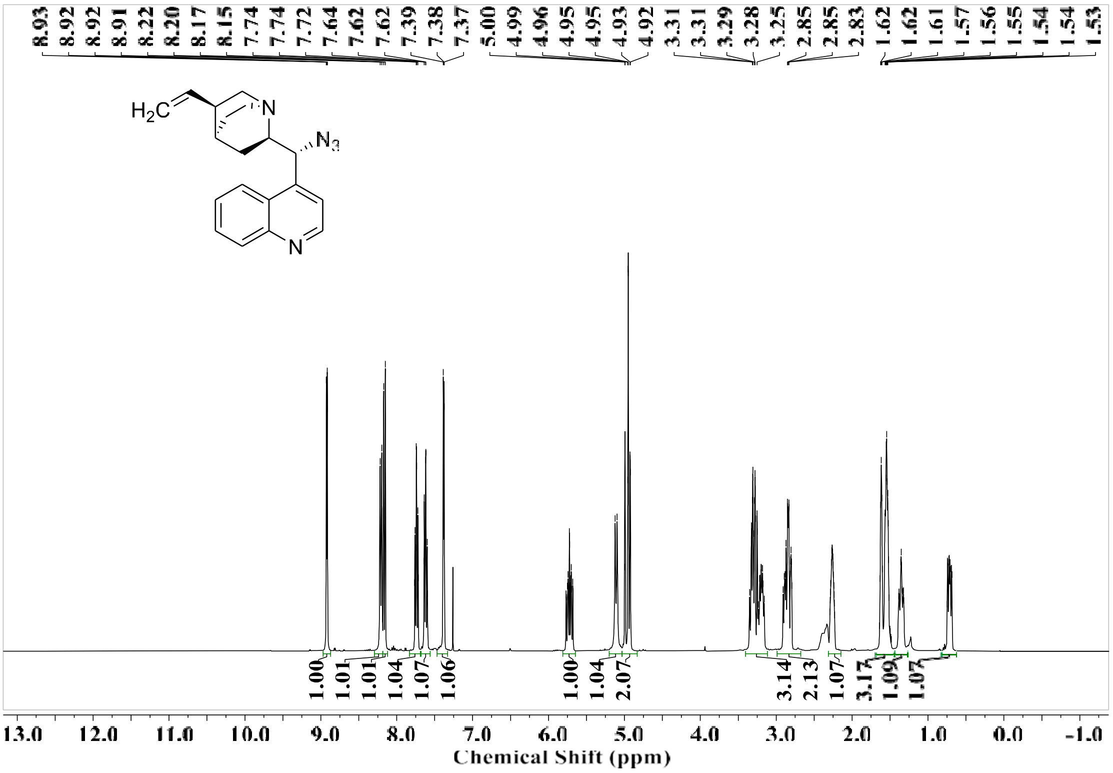

8.93 8.92 8.92 8.91 8.22 8.20 8.17 8.15 7.74 7.74 7.72 7.64 7.62 7.62 7.39 7.38 7.37 5.00 4.99 4.96 4.95 4.95 4.93 4.92 3.31 3.31 3.29 3.28 3.25 2.85 2.85 2.83 1.62 1.62 1.61 1.57 1.56 1.55 1.54 1.54 1.53
H2C
N
N3
N
1.00 1.01 1.01 1.04 1.07 1.06 1.00 1.04 2.07 3.14 2.13 1.07 3.17 1.09 1.07
13.0 12.0 11.0 10.0 9.0 8.0 7.0 6.0 5.0 4.0 3.0 2.0 1.0 0.0 -1.0
Chemical Shift (ppm)

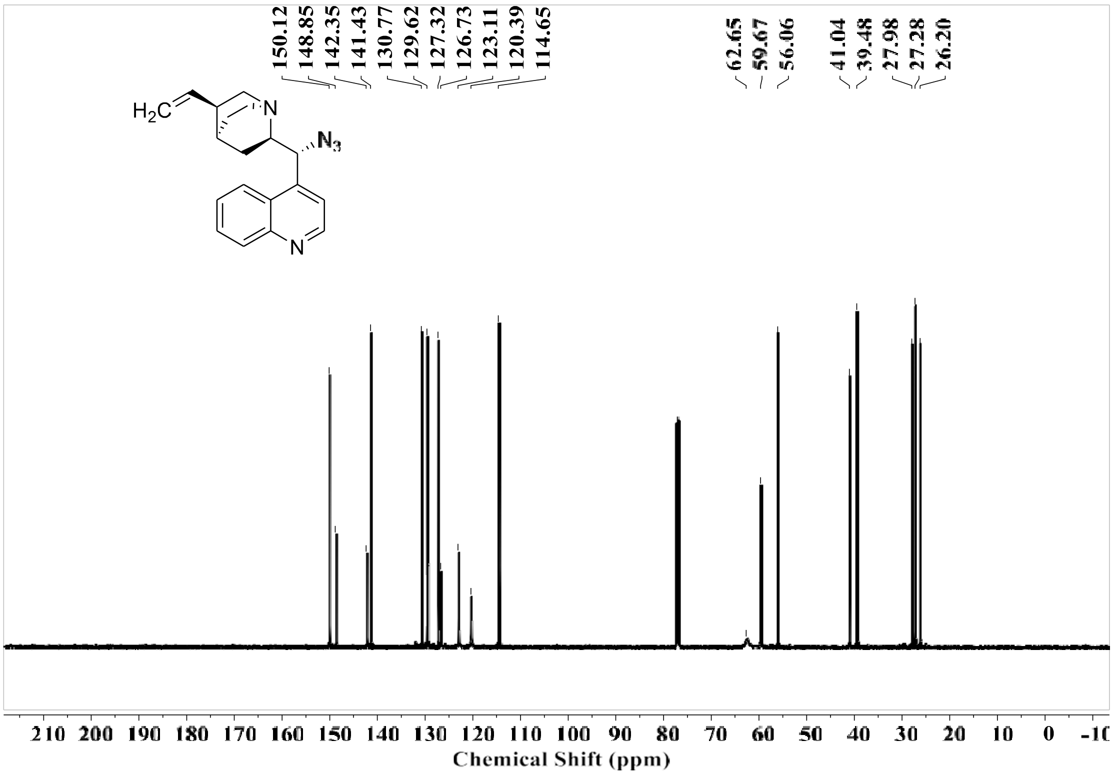

150.12
148.85
142.35
141.43
130.77
129.62
127.32
126.73
123.11
120.39
114.65
62.65
59.67
56.06
41.04
39.48
27.98
27.28
26.20
210 200 190 180 170 160 150 140 130 120 110 100 90 80 70 60 50 40 30 20 10 0 -10
Chemical Shift (ppm)

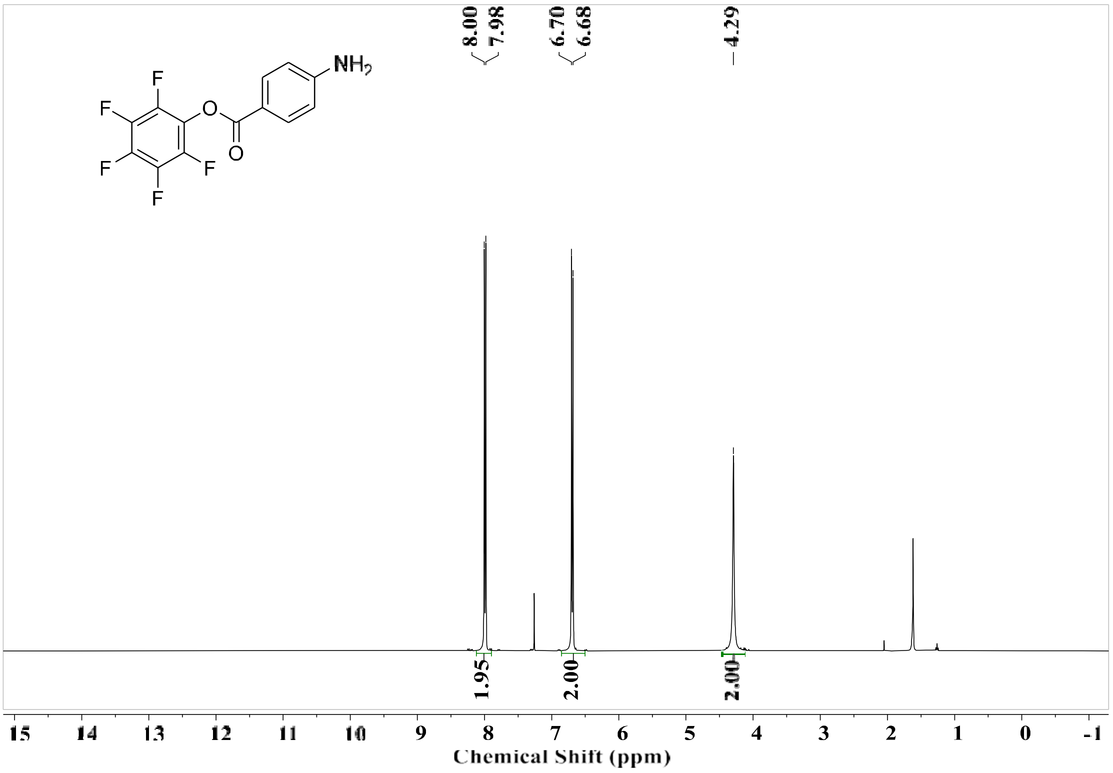

8.00
7.98
6.70
6.68
4.29
1.95
2.00
2.00
15 14 13 12 11 10 9 8 7 6 5 4 3 2 1 0 -1
Chemical Shift (ppm)

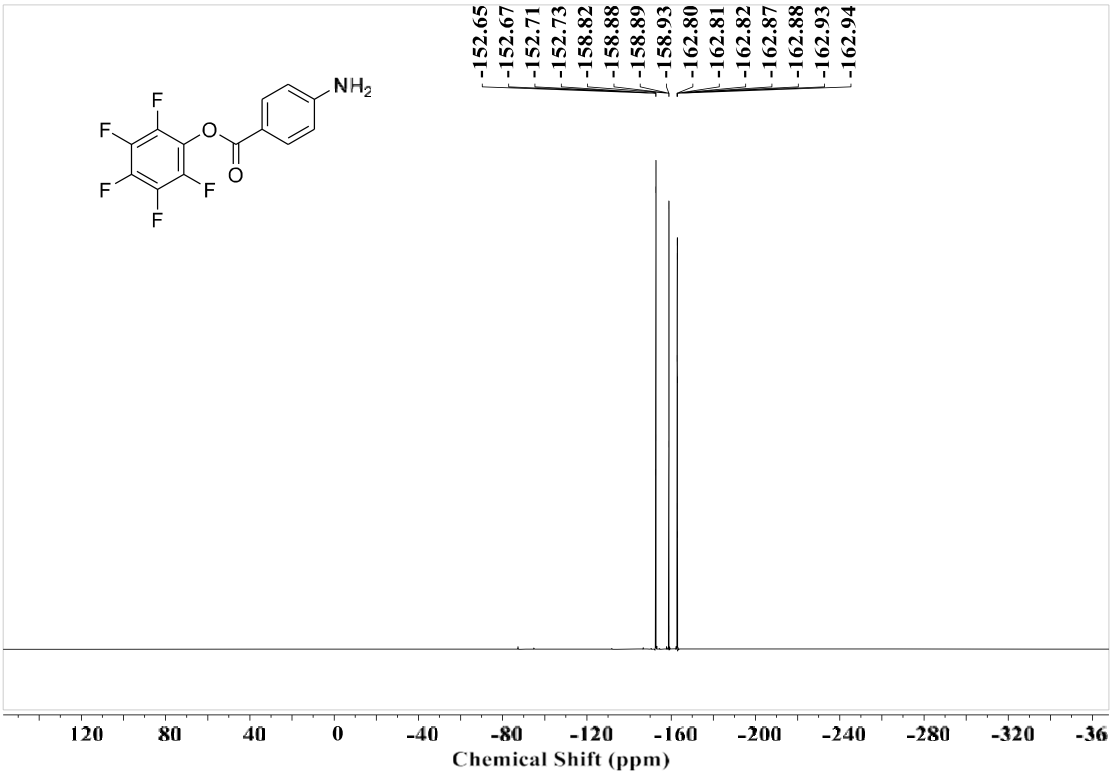
-152.65
-152.67
-152.71
-152.73
-158.82
-158.88
-158.89
-158.93
-162.80
-162.81
-162.82
-162.87
-162.88
-162.93
-162.94
F
F
F
F
F
O
O
NH2
120 80 40 0 -40 -80 -120 -160 -200 -240 -280 -320 -36
Chemical Shift (ppm)

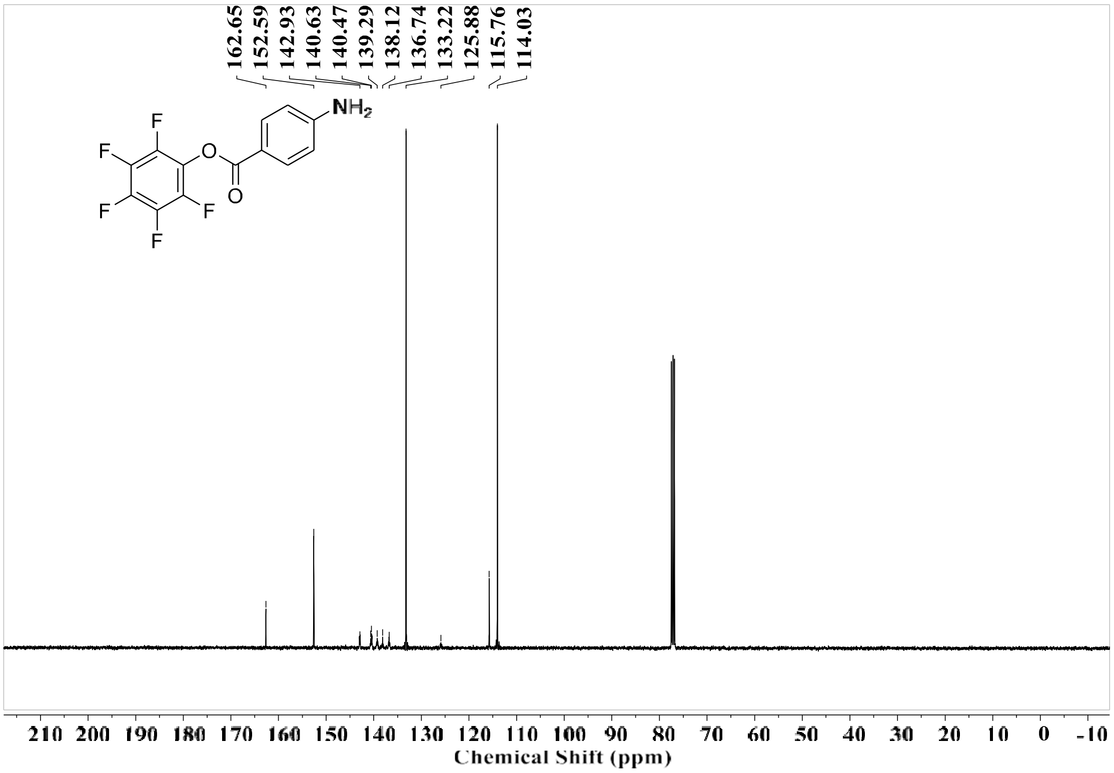
162.65
152.59
142.93
140.63
140.47
139.29
138.12
136.74
133.22
125.88
115.76
114.03
F
F
F
F
F
O
O
NH2
210 200 190 180 170 160 150 140 130 120 110 100 90 80 70 60 50 40 30 20 10 0 -10
Chemical Shift (ppm)

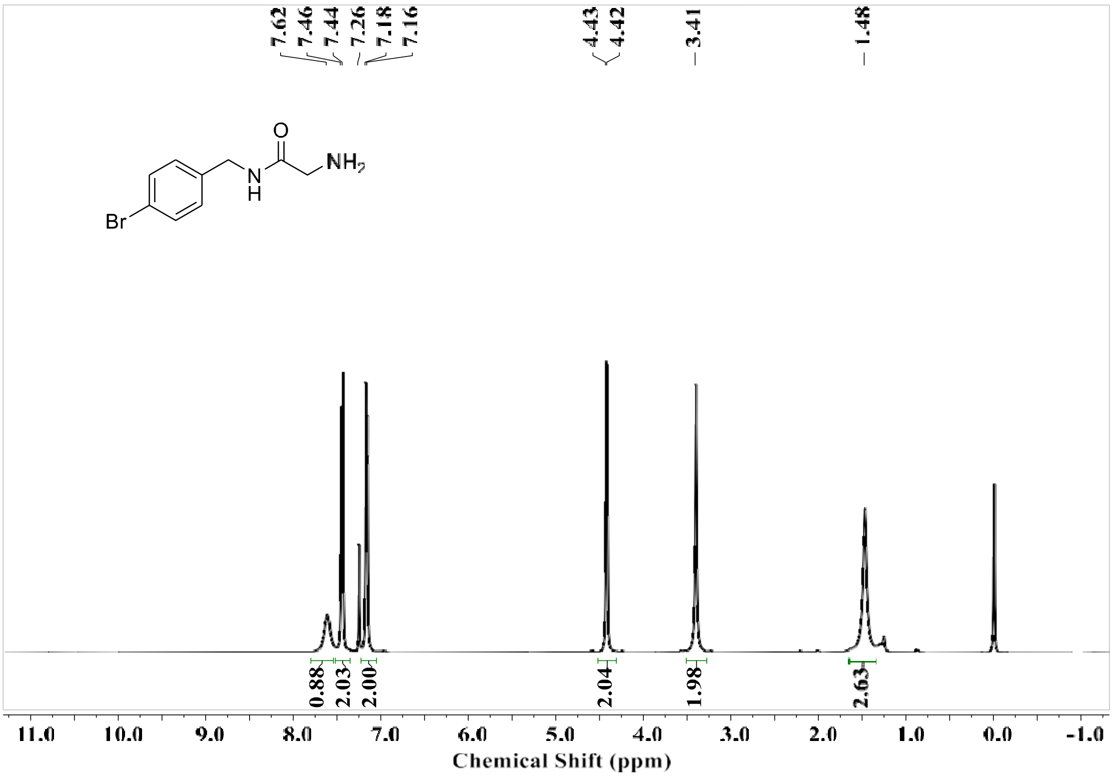
7.62
7.46
7.44
7.26
7.18
7.16
4.43
4.42
3.41
1.48
Br
N
H
O
NH2
0.88
2.03
2.00
2.04
1.98
2.63
11.0 10.0 9.0 8.0 7.0 6.0 5.0 4.0 3.0 2.0 1.0 0.0 -1.0
Chemical Shift (ppm)

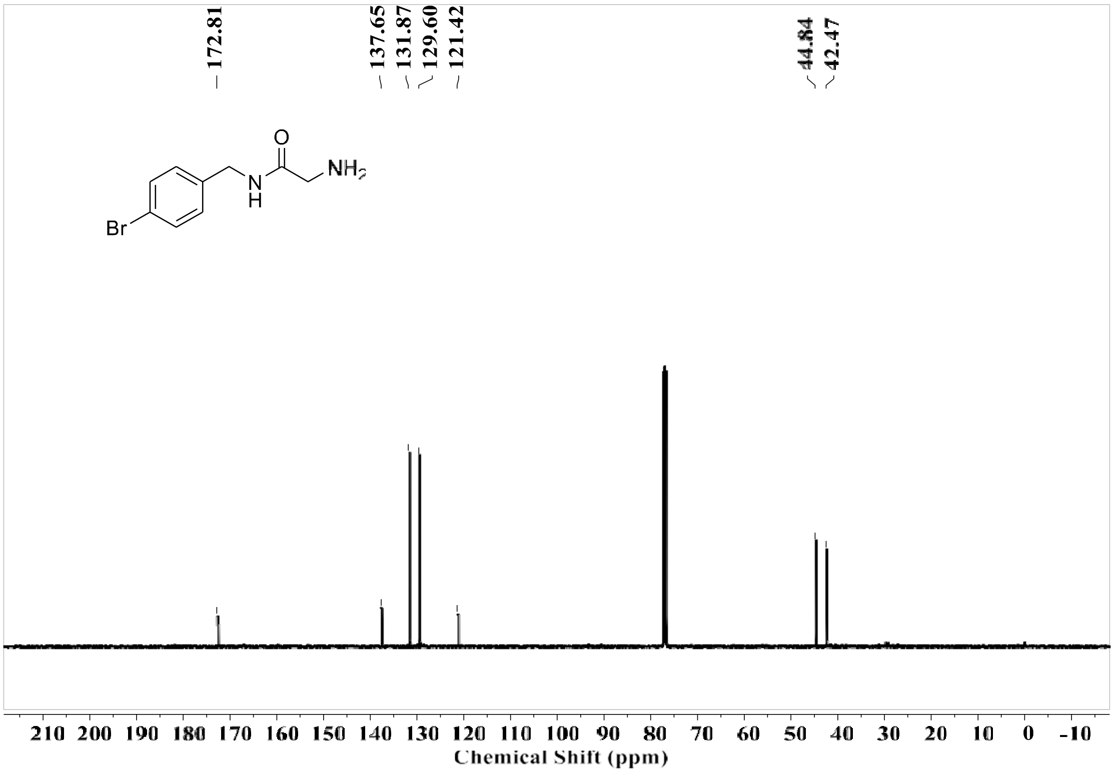
172.81
137.65
131.87
129.60
121.42
44.84
42.47
Br
N
H
O
NH2
210 200 190 180 170 160 150 140 130 120 110 100 90 80 70 60 50 40 30 20 10 0 -10
Chemical Shift (ppm)

3 Reference List for the Characterization Data of the Azides **1** and the Products **3**

Organic azides methyl 4-azidobenzoate **1a**[29,30], 1-azido-4-nitrobenzene **1b**[30], 1-azido-4-(trifluoromethyl)benzene **1c**[30], 1-azido-4-ethynylbenzene **1d**[45]**,** 4-azidobenzenesulfonyl fluoride **1e**[46]**,** 1-azido-4-methoxybenzene **1f**[30], 1-azido-4-iodobenzene **1g**[47]**,** 1-azido-3,5-bis(trifluoromethyl)benzene **1h**[48]**,** methyl 4-azido-2,3,5,6-tetrafluorobenzoate **1i**[48]**,** 1-azido-2-(*tert*-butyl)benzene **1k**[49], 1-azido-2-methylbenzene **1l**[48]**,** 1-azido-3-methoxybenzene **1m**[48], (azidomethylene)dibenzene **1n**[31], (1-azidoethyl)benzene **1o**[46]**,** 2-(azidomethyl)naphthalene **1p**[31], 1-(azidomethyl)-4-nitrobenzene **1q**[31], (4-(azidomethyl)phenyl)(phenyl)methanone **1r**[50], 2-azido-*N*-benzylacetamide **1t**[51], 1-(azidomethyl)pyrene **1u**[52], (2-(4-(azidomethyl)phenyl)ethene-1,1,2-triyl)tribenzene **1v**[53] are known compounds and their analytical data were consistent with literature data.

Methyl 4-aminobenzoate **3a**[54], 4-nitroaniline **3b**[54], 4-(trifluoromethyl)aniline **3c**[54], 4-ethynylaniline **3d**[55], 4-aminobenzenesulfonyl fluoride **3e**[55], 4-methoxyaniline **3f**[50], 4-iodoaniline **3g**[54], 3,5-bis(trifluoromethyl)aniline **3h**[56], methyl 4-amino-2,3,5,6-tetrafluorobenzoate **3i**[57], 2-(*tert*-butyl)aniline **3k**[58], *o*-toluidine **3l**[56], 3-methoxyaniline **3m**[55], diphenylmethanamine **3n**[59], 1-phenylethan-1-amine **3o**[59], naphthalen-2-ylmethanamine **3p**[60], (4-nitrophenyl)methanamine **3q**[61], (4-(aminomethyl)phenyl)(phenyl)methanone **3r**[50], 2-amino-*N*-benzylacetamide **3t**[62], pyren-1-ylmethanamine **3u**[63], (4-(1,2,2-triphenylvinyl)phenyl)methanamine **3v**[64] are known compounds and their analytical data were consistent with literature data.
